# Graded Mulberry Leaf Supplementation Shapes Gut Microbiota, Reprograms Intestinal Metabolism, and Maintains Intestinal Chemical–Immune Barrier Homeostasis in Amur Sturgeon: A Multi-Omics Study

**DOI:** 10.3390/biology15171556

**Published:** 2026-09-05

**Authors:** Wanwan Zhu, Nan Zhang, Xuekai Wang, Yi Xiong, Yongsheng Wang, Fuyu Yang

**Affiliations:** 1 Department of Grassland Science, College of Animal Science, Guizhou University, Guiyang 550025, China; wan850793534@126.com (W.Z.); zn2580042898@126.com (N.Z.); 2 College of Grassland Science and Technology, China Agricultural University, Beijing 100193, China; xkwang2016@163.com; 3 Frontier Technology Research Institute of China Agricultural University in Shenzhen, Shenzhen 518000, China; xiongleslie@126.com; 4 Shenzhen Branch, Guangdong Laboratory of Lingnan Modern Agriculture, Key Laboratory of Livestock and Poultry Multi-Omics of MARA, Agricultural Genomics Institute at Shenzhen, Chinese Academy of Agricultural Sciences, Shenzhen 518000, China

**Keywords:** Amur sturgeon (*Acipenser schrenckii*), mulberry leaf, intestinal barrier homeostasis, gut microbiota, metabolomics, transcriptomics

## Abstract

Mulberry leaf contains abundant phytochemicals with antioxidant and immunomodulatory activities. However, systematic insight into its dose-dependent regulatory effects on the intestinal health of Amur sturgeon remains limited. In the present study, multi-omics approaches, including 16S rRNA gene sequencing, untargeted metabolomics, transcriptomics, together with RT-qPCR, were applied to investigate graded dietary mulberry leaf supplementation in *Acipenser schrenckii*. Juvenile sturgeons were fed four experimental diets containing 0%, 2%, 4% and 6% mulberry leaf over a 10-week feeding trial. Dietary mulberry leaf caused no adverse impacts on growth performance or intestinal digestive capacity. Although the overall structure of the intestinal microbiota remained stable, beneficial bacterial taxa were enriched in a dose-dependent manner. Intestinal metabolism underwent hierarchical remodelling: low inclusion levels supported basal nutrient metabolism, medium inclusion strengthened antioxidant capacity, and high inclusion reprogrammed lipid metabolism and immune function. Mulberry leaf reinforced the intestinal chemical barrier by balancing redox homeostasis and reducing mucosal epithelial permeability. Moreover, intestinal immunity was modulated through three sequential phases: initial innate immune priming, B-cell homing, and the establishment of sustained immune tolerance. In conclusion, mulberry leaf maintains intestinal chemical–immune barrier homeostasis in a dosage-tunable manner, supporting its potential application as a functional aquafeed ingredient.

## Simple Summary

Good intestinal health determines growth performance and disease resistance of farmed fish. Natural plant materials represent attractive feed supplements for sustainable aquaculture, whereas little information is available regarding how different amounts of mulberry leaf regulate intestinal conditions in Amur sturgeon. We conducted a ten-week feeding experiment on juvenile sturgeon using four diets containing graded levels of mulberry leaf. Dietary mulberry leaf exerted no adverse effects on fish growth and digestion. It increased the abundance of beneficial gut bacteria, protected the intestinal barrier, alleviated oxidative stress and regulated intestinal immune responses. Importantly, low, medium and high mulberry leaf inclusion produced distinct metabolic and immune benefits. This study verifies that mulberry leaf can serve as a safe functional feed ingredient to improve intestinal health of Amur sturgeon. The results provide practical guidance for developing environmentally friendly feeding strategies in sturgeon cultivation.

## 1. Introduction

Amur sturgeon (*Acipenser schrenckii*), belonging to the family Acipenseridae, is a precious endemic freshwater fish species in China with extremely high edible and commercial value. As an ancient aquatic species originating approximately 200 million years ago, sturgeons are regarded as classic “living fossils” and serve as an invaluable model for exploring the origin and evolution of vertebrates [1]. Notably, sturgeon caviar, known as “black gold”, represents a globally traded high-end functional aquatic food abundant in nutrients [2]. Intestinal physiological status and homeostatic metabolism are fundamental to nutrient digestion and absorption in sturgeon, thereby influencing overall organism quality and growth performance [3]. In broodstock, the metabolic and nutritional state of the fish is a key determinant of caviar yield and quality, as the biochemical composition of the roe directly shapes the sensory and nutritional properties of this high-value aquaculture commodity [4]. Thus, intestinal health, by modulating the metabolic resources available for reproduction, may indirectly influence caviar quality, highlighting the importance of gut function in the production of sturgeon-derived commodities.

The intestinal chemical–immune barrier forms the core mucosal defense system conserved across aquatic and terrestrial vertebrates. Its homeostasis precisely modulates host–microbe crosstalk, blocks pathogenic invasion, and orchestrates balanced inflammatory responses and antioxidant metabolism [5,6]. Disruption of intestinal barrier function triggered by suboptimal dietary conditions readily induces oxidative stress, persistent mucosal inflammation and gut microecological imbalance. These disturbances further deteriorate organismal health and ultimately alter the nutritional quality of animal-derived foods [7,8]. Under this framework, exploring natural edible plant resources possessing antioxidant, anti-inflammatory and gut-protective capacities facilitates the development of nutritional strategies to stabilize intestinal immune homeostasis and maintain physiological quality of aquatic animals.

Mulberry leaf (*Morus alba* L.) is a well-documented medicinal and edible homologous resource officially recognized by the National Health Commission of the People’s Republic of China [9]. It accumulates abundant natural bioactive phytochemicals, including flavonoids, polysaccharides, and 1-deoxynojirimycin (DNJ), which exert prominent antioxidant, anti-inflammatory and metabolic regulatory effects in multiple biological models [10,11]. China possesses enormous mulberry resources; nevertheless, the vast majority of mulberry leaf biomass remains underexploited, highlighting great potential to develop mulberry leaves as a source of natural dietary bioactive compounds [12]. Accumulating evidence has demonstrated that dietary mulberry leaf (ML) supplementation can improve digestion, antioxidant capacity, and immune performance in aquaculture species. Effective inclusion levels have been shown to range from 4% to 8% across different species, including 4% ML with multi-enzyme premix in golden pompano [13], 4–6% fermented ML in golden pompano [14], and 4–8% processed ML in largemouth bass [15]. Notably, some of those previous studies used enzymatically supplemented or fermented mulberry-leaf products. Despite such differences in raw-material processing, these previously tested levels are broadly comparable to the 2–6% gradient adopted in the present study, supporting the practical relevance of our chosen dosage range. Nevertheless, the molecular mechanisms whereby mulberry leaf bioactives modulate intestinal chemical–immune barrier homeostasis remain poorly defined. In particular, the dose-dependent effects of graded mulberry leaf inclusion on intestinal oxidative equilibrium, inflammatory responses and mucosal barrier integrity in *A. schrenckii* have not yet been clarified. More importantly, an integrated strategy combining 16S rRNA microbiome, transcriptome and untargeted metabolomics coupled with molecular validation has not been adopted to decipher the gut health-regulating mechanisms of mulberry leaves in sturgeons.

In the present study, four isonitrogenous and isoenergetic diets containing graded inclusion levels of mulberry leaf were formulated. We comprehensively evaluated intestinal histomorphology, digestive enzyme activity, intestinal permeability, antioxidant capacity and gut microbiota composition, together with transcriptomic and metabolomic profiles, and verified key functional pathways via qPCR. This study aimed to elucidate the multi-omics mechanisms through which mulberry leaf bioactives sustain intestinal chemical–immune barrier homeostasis by remodeling gut microecology and coordinating antioxidant and immune balance. Our findings reveal, for the first time, the dose-dependent multi-omics pathways through which mulberry leaf bioactives orchestrate intestinal chemical–immune barrier homeostasis in sturgeon. These results not only advance our understanding of mucosal immune regulation in basal ray-finned fishes but also provide a mechanistic basis for developing mulberry leaves as a functional additive to safeguard the physiological quality of sturgeon-derived aquatic products.

## 2. Materials and Methods

### 2.1. Animal Ethics

All experiments were approved by the Inspection Form for the Guizhou University Experimental Animal Ethics (approval No. EAE-GZU-20240E072; approval date: 31 December 2024). All animal manipulations were conducted in accordance with the ARRIVE 2.0 guidelines.

### 2.2. Experimental Diets

Mulberry leaves (ML) applied in diet preparation were purchased from Shichao Fruit Wood Planting Farmers Professional Cooperative (Shangqiu, Henan, China). The ML were harvested in June, naturally air-dried at ambient temperature until constant dry matter weight was achieved, and then ground. The detailed proximate composition and bioactive constituents of the ML powder were determined prior to feed formulation, and the complete characterization data are presented in Table S1.

Prior to feed formulation, the proximate compositions of all dietary ingredients were calculated based on the values declared by the manufacturers. Using these data and the published nutrient and amino acid requirements of Amur sturgeon (*A. schrenckii*) [16,17], we calculated the inclusion levels of ML that would allow all experimental diets to remain isonitrogenous and isoenergetic. Through iterative formulation calculations, 6% ML inclusion was identified as the maximum level that could be incorporated while still meeting both the protein and energy targets. Accordingly, four experimental diets were formulated with graded ML levels: CK (0% ML), ML2 (2% ML), ML4 (4% ML), and ML6 (6% ML). To maintain isonitrogenous and isoenergetic conditions, the proportions of fishmeal and wheat middling were reduced as ML inclusion increased, with adjustments to wheat gluten meal, fish oil, and crystalline amino acids (L-Lysine, DL-Methionine, and Threonine) to balance nutrient profiles. The complete feed formulas and determined nutritional compositions are presented in Table S2, and the results confirmed that all diets were successfully isonitrogenous and isoenergetic.

### 2.3. Animals and Rearing Conditions

A total of 600 juvenile Amur sturgeons (*A. schrenckii*) were sourced from the Beijing Amur Sturgeon Elite Breeding Base (Beijing, China). The experimental fish were sexually undifferentiated individuals with no genetic modification and had not received any experimental treatments before this trial. All fish were subjected to a 4-week acclimation period under standardized rearing conditions before the feeding trial commenced. Following 24 h feed deprivation, 320 healthy fish of mixed sex with uniform initial body weight (IBW = 198.64 ± 0.99 g) were haphazardly selected and allocated to four dietary treatments, with four biological replicates per treatment. Each replicate was housed in an independent net cage holding 20 fish. Fish were hand-fed twice daily to apparent satiation throughout the 10-week feeding trial, and uneaten feed was collected to evaluate feed intake. Water quality parameters were stably controlled: water temperature 20 ± 2 °C, pH 7.5–8.5, dissolved oxygen ≥ 7.0 mg/L. Fish health status was monitored daily; predefined humane endpoints were applied for individuals exhibiting severe stress, injury or appetite loss.

### 2.4. Sample and Data Collection

Feed intake, mortality and body weight were recorded throughout the feeding trial. All fish were deprived of feed for 24 h prior to tissue sampling. Twelve fish per dietary treatment (3 fish randomly selected from each of the 4 replicate tanks) were sampled. These 12 fish constituted the total sample pool for all subsequent analyses; different subsets of this pool were allocated to specific assays, with the relationships being nested rather than parallel. Fish were anesthetized using buffered MS-222 (100 mg/L, tricaine methanesulfonate; Ablome, Shanghai, China). After recording final body weight and body length, fish were humanely euthanized under deep anesthesia. Liver and visceral mass were weighed to calculate somatic growth indices.

Blood samples were collected from the caudal vein. Whole blood was allowed to clot for 30 min at room temperature, followed by centrifugation at 3500× *g* for 10 min at 4 °C; separated serum was preserved at −80 °C for subsequent biochemical assays. Foregut segments were snap-frozen in liquid nitrogen and stored at −80 °C for digestive enzyme determination. Segments of foregut (approximately 2 cm in length) were fixed in 4% paraformaldehyde for histological analysis. Midgut and hindgut tissues were immersed in RNALater™ (Cat. No. R0118FT; Beyotime Institute of Biotechnology, Shanghai, China), incubated overnight at 4 °C, and then stored at −80 °C for total RNA extraction. Additional midgut tissue samples were snap-frozen and cryopreserved at −80 °C for untargeted metabolomics. Gut tissue together with intestinal digesta was collected and preserved at −80 °C for gut microbiota profiling.

At the fish allocation stage and during daily feeding and routine management, the researchers responsible for husbandry knew the group allocation, as the experimental diets differed visually, making blinding impractical during feeding. During sampling, tissue dissection, and laboratory measurements, all samples were labelled only with serial numbers without treatment information. Operators performing the laboratory analyses were unaware of group identity. Thus, while complete blinding was not feasible during the feeding phase, all subsequent sample processing and data acquisition were performed under blinded conditions.

### 2.5. Growth Performance

A set of somatic and growth indicators was calculated to assess the growth status of Amur sturgeon, including weight gain rate (WGR), specific growth rate (SGR), feed conversion ratio (FCR), feed rate (FR), survival rate (SR), condition factor (CF), hepatosomatic index (HSI) and viscerosomatic index (VSI). All calculations were performed based on replicate-level data. The corresponding computational formulas are presented in Table S3.

### 2.6. Biochemistry Indices

Serum biochemical and antioxidant parameters, including total antioxidant capacity (T-AOC; Cat. No. A015-2), superoxide dismutase (SOD; Cat. No. A001-1) and malondialdehyde (MDA; Cat. No. A003-1), were determined using commercial assay kits from Nanjing Jiancheng Bioengineering Institute (Nanjing, China). Serum immunoglobulin M (IgM; Cat. No. IG7437), complement 3 (C3; Cat. No. C37392), complement 4 (C4; Cat. No. C47402) and alkaline phosphatase (ALP; Cat. No. AP6030) were quantified with kits provided by Beijing Leadman Biochemical Co., Ltd. (Beijing, China). Intestinal digestive enzyme activities, namely amylase (AMS; Cat. No.C016-1-1), trypsin (Cat. No.A080-2-1) and lipase (LPS; Cat. No. A054-2-1), were measured using kits from Nanjing Jiancheng Bioengineering Institute (Nanjing, China). Diamine oxidase (DAO; Cat. No. YZ-040115) activity was assayed with commercial kits obtained from Bioyappp Technology Co., Ltd. (Shanghai, China).

### 2.7. Intestinal Histological Sectioning

Following fixation, intestinal tissue samples were trimmed, dehydrated, paraffin-embedded, sectioned, stained and mounted onto glass slides. Histological slides were scanned using a Pannoramic digital slide scanner (3DHISTECH Ltd., Budapest, Hungary). Villus height (Vh) and crypt depth (Cd) were visualized and quantified with CaseViewer 2.4 (3DHISTECH Ltd., Budapest, Hungary) and Image Pro Plus 6.0 software (Media Cybernetics, Rockville, MD, USA). For each histological section, goblet cells were counted within three randomly selected visual fields at 200× magnification. Goblet cell density was calculated as the goblet cell number divided by the area of the corresponding microscopic field.

### 2.8. Composition and Diversity of Intestinal Microbiota

Total microbial DNA was extracted from homogenized intestinal samples using the MagBeads FastDNA Kit for Soil (Cat. No. 11656438, MP Biomedicals, Santa Ana, CA, USA). DNA concentration and purity were assessed using a NanoDrop spectrophotometer (Thermo Fisher Scientific, Waltham, MA, USA). The V3–V4 hypervariable region of the bacterial 16S rRNA gene was amplified, and paired-end 2 × 250 bp sequencing was performed on the Illumina NovaSeq 6000 platform (Illumina, San Diego, CA, USA) using a 500-cycle SP kit.

All bioinformatic analyses were implemented in QIIME2 v2024.5. Raw reads were subjected to quality filtering, denoising, read merging and chimera removal using the DADA2 plugin [18,19]. Alpha diversity indices (Chao1, Observed species, Shannon, Simpson) were calculated to characterize community richness and evenness. For β-diversity analysis, Bray–Curtis distances were computed for non-metric multidimensional scaling (NMDS) to visualize dissimilarities in microbial communities. Permdisp was adopted to test homogeneity of dispersion, and permutational multivariate analysis of variance (PERMANOVA) was used to identify significant intergroup differences in community structure. Differential taxa were screened using linear discriminant analysis effect size (LEfSe), integrating the Kruskal–Wallis H test, pairwise Wilcoxon test and linear discriminant analysis (LDA), following the original implementation of this method. As an exploratory analysis, we did not apply multiple-testing correction; instead, we adopted a stringent LDA-score threshold (>3) to filter taxa with robust effect sizes. Taxa with an LDA score > 3 and *p* < 0.05 were regarded as potential biomarkers requiring further validation.

### 2.9. Intestinal Untargeted Metabolomics Analysis

Intestinal tissues were snap-frozen in liquid nitrogen and stored at −80 °C prior to metabolite extraction. Metabolites were separated using a Thermo Vanquish Flex UHPLC system (Thermo Fisher Scientific, Waltham, MA, USA) coupled to an Orbitrap Exploris 120 mass spectrometer (Thermo Fisher Scientific, Waltham, MA, USA) equipped with an HESI ion source. Data acquisition was carried out in both positive and negative electrospray ion modes. Chromatographic separation was achieved on an ACQUITY UPLC HSS T3 column (1.8 μm, 2.1 mm × 100 mm; Waters Corporation, Milford, MA, USA). The chromatographic parameters were set as follows: flow rate 0.4 mL/min, column temperature 40 °C, autosampler temperature 8 °C, injection volume 2 μL. Mobile phase A consisted of 0.1% aqueous formic acid, and mobile phase B was acetonitrile containing 0.1% formic acid (*v*/*v*). Mass spectrometry data were acquired under data-dependent acquisition (DDA) mode controlled by Xcalibur v4.7 (Thermo Fisher Scientific, Waltham, MA, USA). Major MS parameters: spray voltage 3.5 kV (positive)/−3.0 kV (negative), sheath gas 40 arb, auxiliary gas 10 arb, capillary temperature 320 °C, auxiliary gas heater temperature 300 °C; full-scan resolution set to 60,000 within the m/z range of 70–1000. The top four precursor ions were fragmented by HCD at normalized collision energy of 30%, with a resolution of 15,000; dynamic exclusion duration was set to 4 s. Automatic gain control (AGC) and maximum injection time (Max IT) were maintained at default settings.

Raw metabolomic data were processed in MS-DIAL v4.9.221218 for peak picking, alignment and metabolite annotation. Features with missing values exceeding 50% within each group were discarded. Following missing value imputation and data normalization, metabolites with a relative standard deviation (RSD) > 30% in quality control (QC) samples were filtered out. Principal component analysis (PCA) was implemented in R v4.3.3 to evaluate analytical repeatability. Orthogonal partial least squares discriminant analysis (OPLS-DA) combined with two-tailed Student’s *t*-test was used to screen differential metabolites. In this exploratory untargeted metabolomic screening, FDR-adjusted *p*-values were calculated via the Benjamini–Hochberg method, but initial differential metabolite selection used uncorrected *p* < 0.05 rather than FDR thresholds. Given that stringent FDR correction may discard moderate-effect metabolites with biological relevance in exploratory studies, we adopted a combined filter of PLS-DA VIP > 1, |FC| > 1, and uncorrected *p* < 0.05 to identify candidate metabolites, balancing biological retention against false-positive risks from single-threshold reliance. These identified metabolites are considered exploratory differential candidates requiring further validation. Kyoto Encyclopedia of Genes and Genomes (KEGG) enrichment analysis of differential metabolites was performed using clusterProfiler v4.6.0.

### 2.10. Intestinal Transcriptome Analysis

Intestinal tissues were snap-frozen in liquid nitrogen and stored at −80 °C. Total RNA was extracted using TRIzol reagent (Invitrogen, Carlsbad, CA, USA). RNA concentration and purity were determined with a NanoDrop One spectrophotometer (Thermo Fisher Scientific, Waltham, MA, USA), while RNA integrity was assessed by agarose gel electrophoresis and an Agilent Fragment Analyzer 5400 system (Agilent Technologies, Santa Clara, CA, USA). Qualified RNA samples were subjected to mRNA enrichment, fragmentation, reverse transcription, end repair, A-tailing and adapter ligation for cDNA library construction. Libraries were sequenced on the Illumina NovaSeq X Plus platform (Illumina, San Diego, CA, USA).

Raw sequencing reads were quality-trimmed using Fastp v0.22.0 to generate clean reads, which were mapped against the reference genome with HISAT2 v2.1.0. Gene expression levels were normalized to fragments per kilobase of transcript per million mapped reads (FPKM). Differentially expressed genes (DEGs) were identified using DESeq2 v1.38.3 with thresholds of |log_2_FC| ≥ 1 and *p* < 0.05. Clustering heatmaps of DEGs were visualized via ComplexHeatmap v2.16.0. Gene Ontology (GO) and Kyoto Encyclopedia of Genes and Genomes (KEGG) enrichment analyses for DEGs were conducted using clusterProfiler v4.6.0.

### 2.11. Correlation Analysis of Multi-Omics Data

Spearman rank correlation analysis was performed to evaluate the associations between genus-level differential gut microbes and differential metabolites. Correlation pairs with *p* < 0.05 and an absolute correlation coefficient > 0.5 were considered statistically significant. The top 20 microbe–metabolite correlation pairs were visualized in a clustered heatmap, in which red indicates positive correlations and blue–purple indicates negative correlations, with asterisks denoting significant correlations. Pearson correlation analysis was further applied to explore the regulatory relationships between differentially expressed genes and differential metabolites. Gene–metabolite pairs with *p* < 0.05 were identified as significantly correlated, and the top 20 correlation pairs were displayed in a heatmap to characterize the overall transcriptome–metabolome interaction patterns.

### 2.12. RNA Isolation, Reverse Transcription, and mRNA Level Analysis

Intestinal total RNA was extracted using the RNA EasyFast Kit (Tiangen Biotech, Beijing, China). RNA integrity was verified by 1% agarose gel electrophoresis, while RNA concentration and purity (OD260/280 ratio) were determined with a NanoDrop 2000 spectrophotometer (Thermo Fisher Scientific, Waltham, MA, USA). The *β-actin* gene (GenBank: AY649619) was used as the internal reference gene for the normalization of target gene expression levels.

Partial quantitative real-time PCR (RT-qPCR) primers for *A. schrenckii* were retrieved from previously published literature [20]. The remaining target primers were designed using SnapGene 6.0.2 (Dotmatics, Boston, MA, USA) based on the transcriptome sequencing data obtained in the present study. All primer sequences are summarized in Supplementary Table S4. RT-qPCR amplification was performed on a qTOWER Iris real-time PCR system (Analytik Jena, Jena, Germany) with TB Green^®^ Premix Ex Taq™ II (Takara Bio, Shiga, Japan). Each sample was analyzed with three independent biological replicates.

### 2.13. Statistical Analysis

All statistical analyses for phenotypic data were performed using GraphPad Prism 10.6.0 (San Diego, CA, USA). Prior to parametric testing, data normality and homogeneity of variances were verified. When parametric assumptions were violated, appropriate non-parametric tests were adopted. One-way analysis of variance (one-way ANOVA) was used to evaluate growth indices, serum biochemical parameters, intestinal morphological traits and digestive enzyme activities, followed by Tukey’s post hoc test for multiple pairwise comparisons among treatments. All phenotypic data are presented as mean ± standard error of the mean (SEM). Statistical significance was set at *p* < 0.05.

For variables showing significant intergroup differences in one-way ANOVA (*p* < 0.05), linear and quadratic polynomial regression models were used to characterize dose–response relationships between graded dietary mulberry leaf supplementation and measured phenotypes. Model selection was determined based on the significance of linear (*p_linear_*) and quadratic (*p_quadratic_*) terms. The linear model was retained if only the linear term reached significance; the quadratic model was selected when the quadratic term was significant. No clear dose-dependent trend was considered to exist if neither linear nor quadratic terms were statistically significant.

Multi-omics correlation analyses were conducted in R v4.3.3, as described in corresponding sections above.

## 3. Results

### 3.1. Growth Performance and Morphological Parameters

No significant differences in final body weight (FBW), survival rate (SR), weight gain rate (WGR), specific growth rate (SGR), feeding rate (FR), condition factor (CF), and viscerosomatic index (VSI) were observed among all mulberry leaf supplementation groups compared with the control group (*p* > 0.05). The hepatosomatic index (HSI) was significantly decreased in the ML6 group relative to the control group (*p* < 0.05). Additionally, HSI exhibited a significant linear decreasing trend with increasing dietary mulberry leaf inclusion levels (*p_linear_* < 0.05). Detailed data regarding growth and morphological parameters are provided in Table S5.

### 3.2. Changes in Serum Antioxidant-Related Indices and Immune Parameters

**Figure 1:**
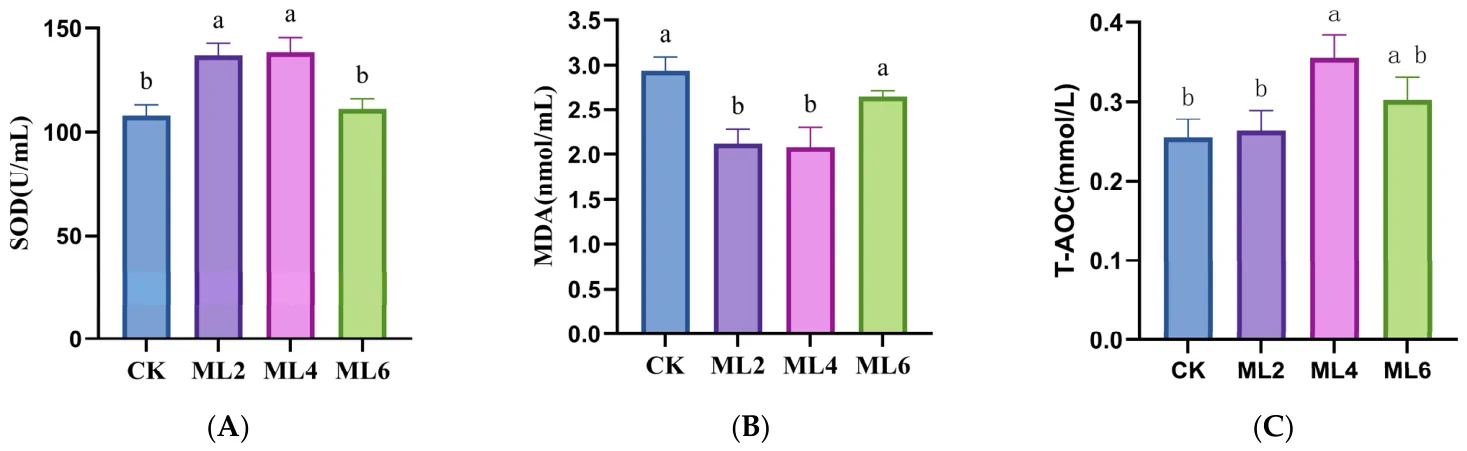

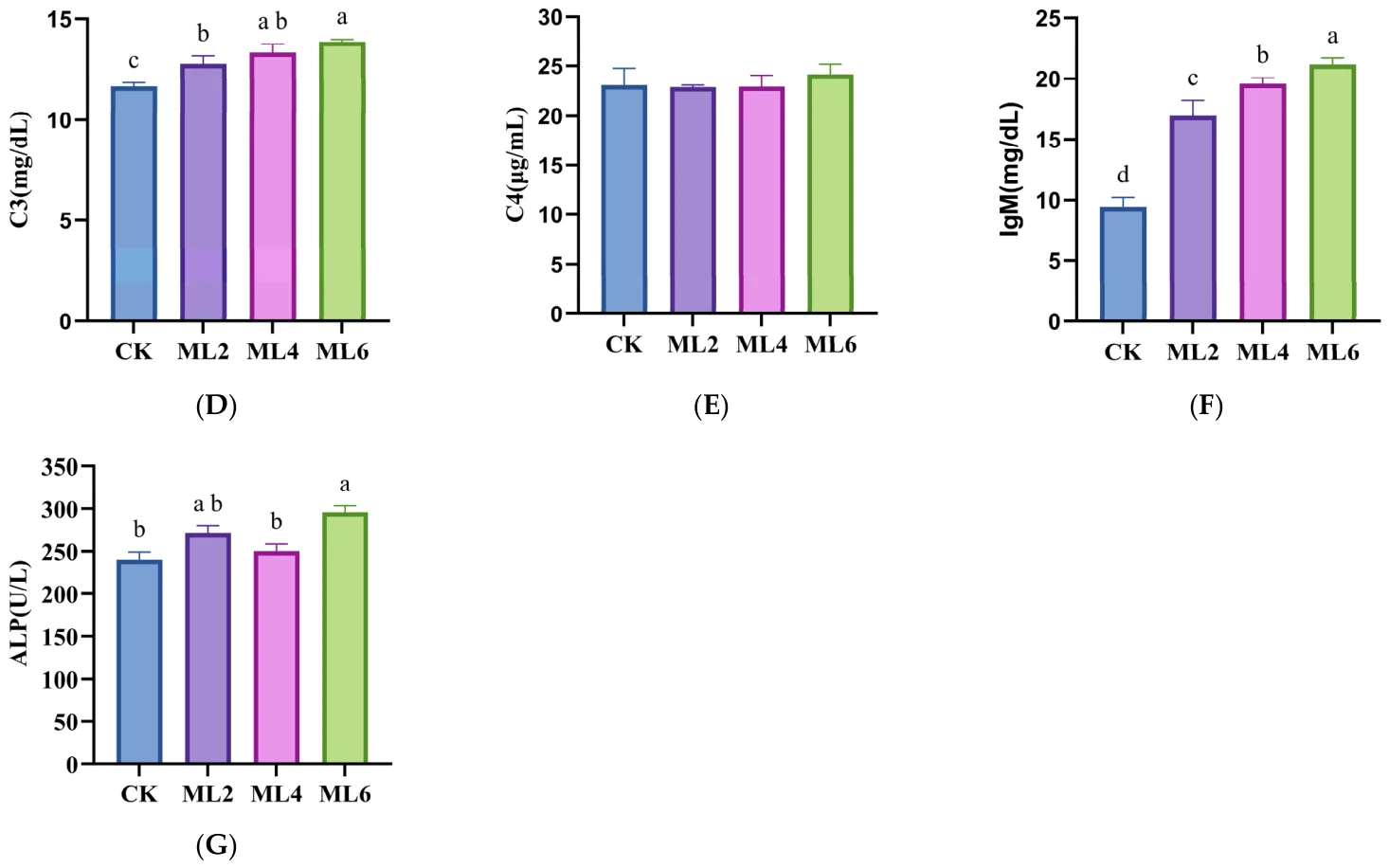
Effects of dietary ML supplementation on serum antioxidant indices and immune parameters of *A. schrenckii* (*n* = 3). (**A**–**C**) Serum antioxidant indicators: (**A**) SOD activity; (**B**) MDA content; (**C**) T-AOC level. (**D**–**G**) Serum immune indices: (**D**) C3 concentration; (**E**) C4 concentration; (**F**) IgM concentration; (**G**) ALP activity. All data are expressed as mean ± SEM. Columns marked with distinct lowercase letters are significantly different among groups (*p* < 0.05).

Compared with the CK group, significantly higher SOD activity and lower MDA content were observed in the ML2 and ML4 groups (*p* < 0.05). Quadratic polynomial regression analysis confirmed significant quadratic responses of SOD and MDA to graded mulberry leaf supplementation (*p_quadratic_* < 0.05). Specifically, SOD activity increased initially and then decreased, reaching the maximum value at a mulberry leaf inclusion level of 3.08% (Figure 1A). By contrast, MDA content decreased first and then increased, with the minimum value appearing at the inclusion level of 3.26% (Figure 1B). The total antioxidant capacity (T-AOC) was significantly enhanced in the ML4 group and showed a significant linear increasing trend with the elevation of dietary mulberry leaf levels (*p_linear_* < 0.05) (Figure 1C).

All mulberry leaf supplementation groups exhibited significantly higher C3 concentrations than the control group (*p* < 0.05), with a distinct linear increasing trend along with rising mulberry leaf inclusion (*p_linear_* < 0.05) (Figure 1D). No significant differences in C4 concentrations were detected among all groups (*p* > 0.05) (Figure 1E). Serum IgM levels were markedly upregulated in all treatment groups (*p* < 0.05) and fitted a significant quadratic regression model with a slowed growth rate, peaking at the inclusion level of 5.58% (*p_linear_* < 0.05, *p_quadratic_* < 0.05) (Figure 1F). Additionally, ALP activity was significantly higher in the ML6 group (*p* < 0.05) and presented a significant linear upward trend with increasing dietary mulberry leaf dosage (*p_linear_* < 0.05) (Figure 1G).

### 3.3. Changes in Intestinal Digestive Function, Barrier-Related Indices and Intestinal Morphology

**Figure 2:**
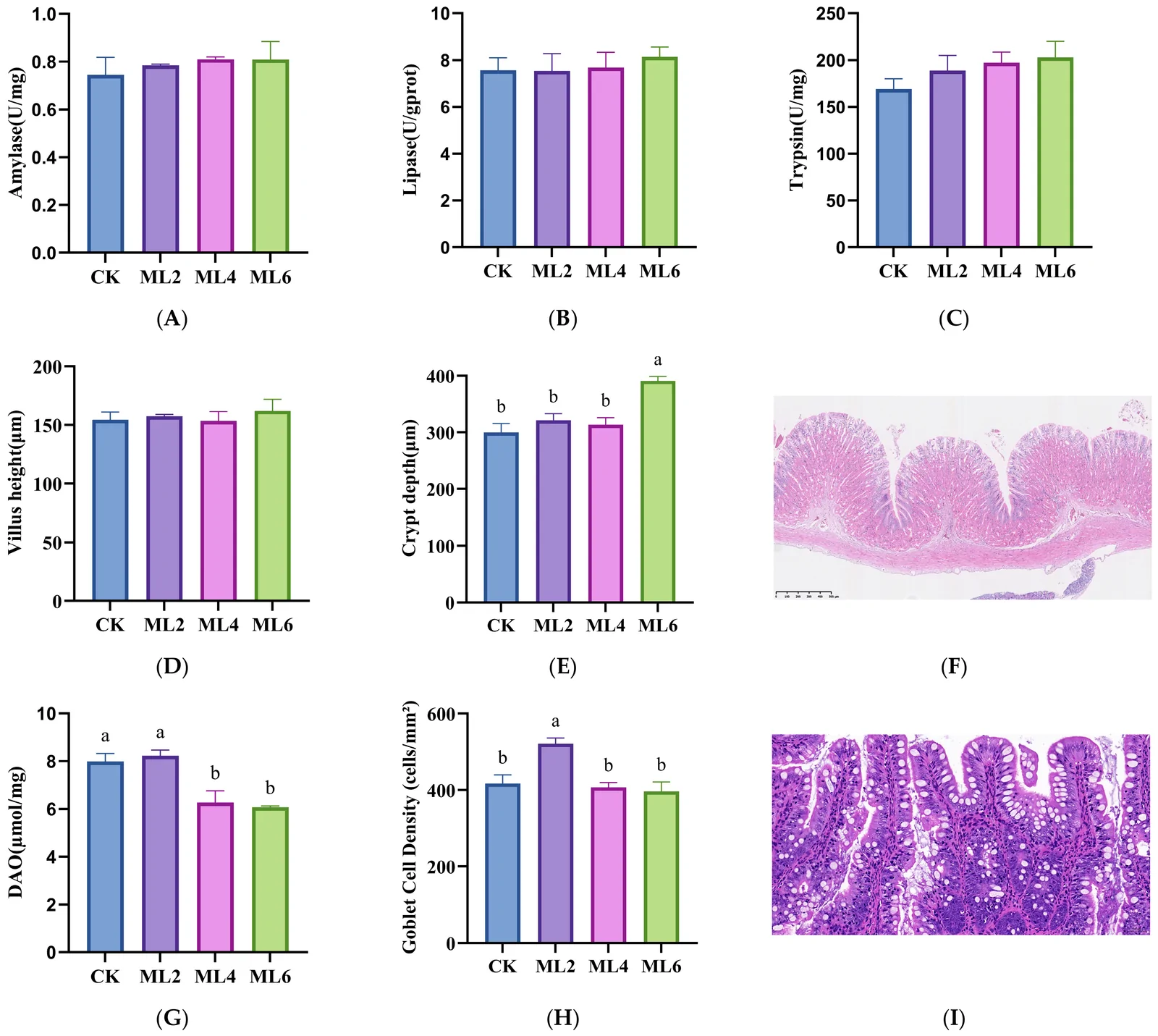
Effects of dietary ML supplementation on intestinal digestive enzymes, morphology, barrier function and goblet cell number of *A. schrenckii* (*n* = 3). (**A**–**C**) Intestinal digestive enzymes: (**A**) amylase activity; (**B**) lipase activity; (**C**) trypsin activity. (**D**–**F**) Intestinal morphological indexes: (**D**) villus height; (**E**) crypt depth; (**F**) representative HE histological micrographs of intestinal tissue. (**G**) DAO activity; (**H**) goblet cell density; (**I**) representative histological images of goblet cell staining. Data are presented as mean ± SEM. Values with different lowercase letters indicate significant differences between groups (*p* < 0.05).

No significant differences in intestinal amylase, lipase, and trypsin activities, as well as intestinal villus height, were found across all experimental groups (*p* > 0.05) (Figure 2A–D). The crypt depth was significantly increased in the ML6 group compared with the CK group (*p* < 0.05). Both linear and quadratic regression analyses revealed significant dose-dependent responses (*p_linear_* = 0.0014, *p_quadratic_* = 0.0308), with the inflection point calculated at a mulberry leaf inclusion level of 1.09%. Overall, crypt depth increased gradually with the elevation of dietary mulberry leaf supplementation within the tested dosage range (Figure 2E). Representative histological images of intestinal tissue sections are presented in Figure 2F.

Intestinal DAO activity was significantly decreased in the ML4 and ML6 groups (*p* < 0.05) and exhibited a distinct linear declining trend with increasing mulberry leaf dosage (*p_linear_* < 0.05) (Figure 2G). The number of intestinal goblet cells was markedly higher in the ML2 group relative to the control (*p* < 0.05), whereas no significant linear or quadratic dose–response relationships were observed among all treatments (*p* > 0.05) (Figure 2H). Representative histological images for goblet cell quantification are shown in Figure 2I.

### 3.4. Intestinal Microbial Community Composition and Diversity of A. schrenckii

**Figure 3:**
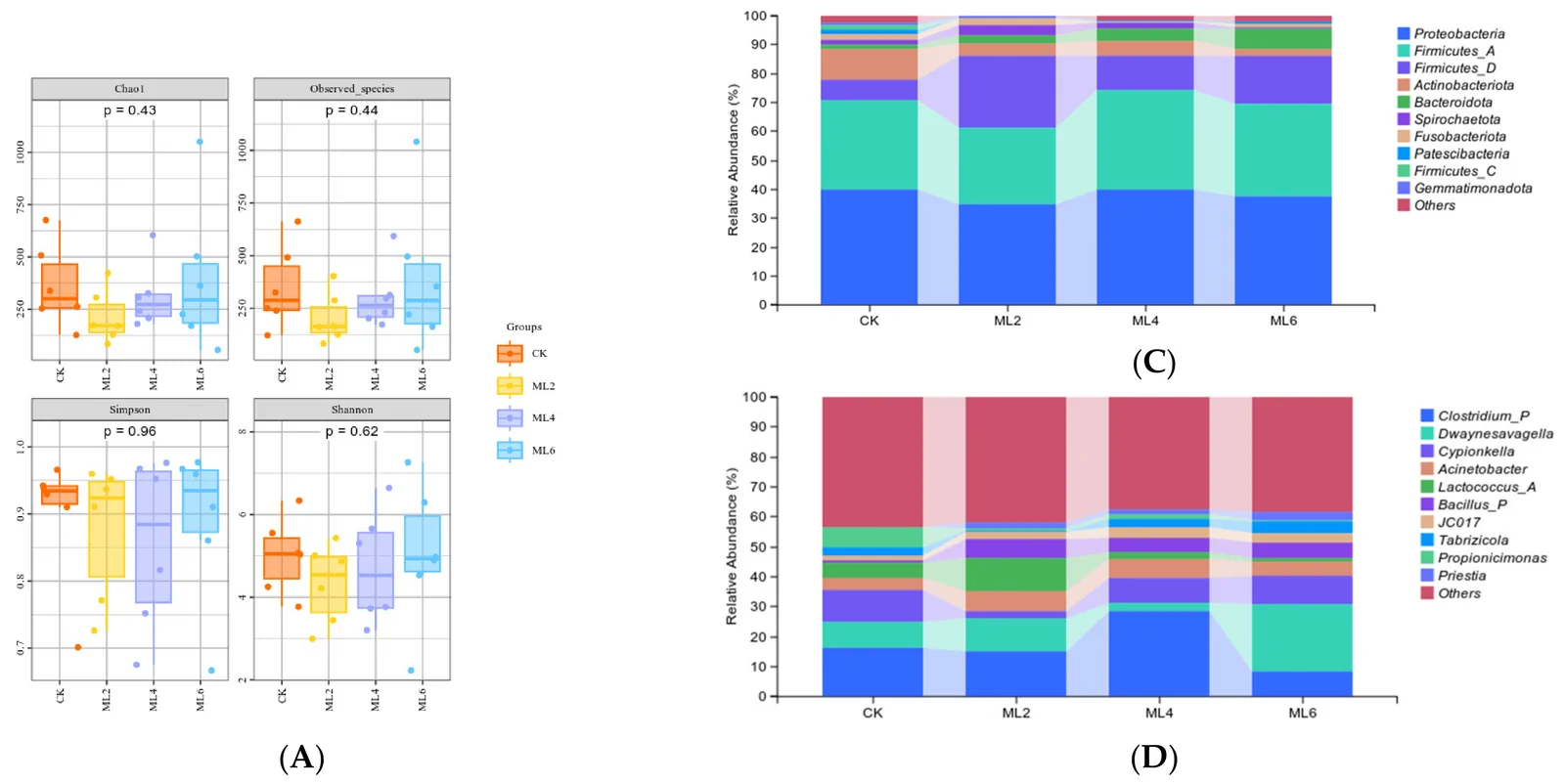

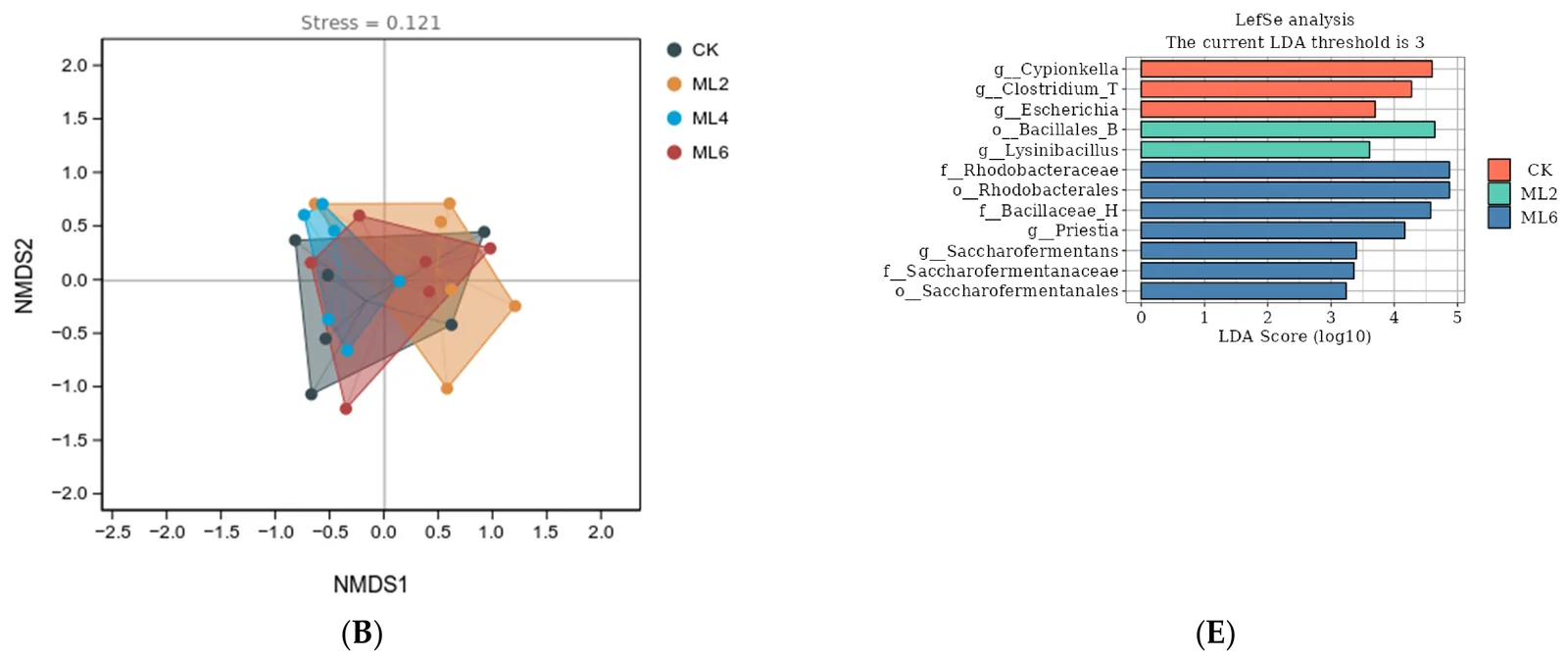
Effects of ML on intestinal microbial diversity and community composition (*n* = 6). (**A**) Alpha-diversity indices of intestinal microbiota among different groups. (**B**) Beta-diversity revealed by Bray–Curtis distance-based NMDS analysis. (**C**) Microbial community composition at the phylum level. (**D**) Microbial community composition at the genus level. (**E**) Group-specific differential microbial biomarkers identified by LEfSe analysis.

The effects of dietary mulberry leaf (ML) supplementation on intestinal microbial diversity and community structure of *A. schrenckii* were evaluated via 16S rRNA gene sequencing. No significant differences in four typical alpha-diversity indices were observed among all treatment groups (*p* > 0.05) (Figure 3A). For beta-diversity analysis, non-metric multidimensional scaling (NMDS) based on Bray–Curtis distances produced a stress value of 0.121 (<0.2), indicating reliable and valid ordination results (Figure 3B). Permdisp analysis yielded *p* = 0.296 (*p* > 0.05), confirming homogeneous intra-group microbial dispersion across all dietary treatments. Furthermore, PERMANOVA revealed no significant overall alterations in gut microbial community structure among groups (*p* = 0.28, *p* > 0.05). Collectively, dietary ML supplementation did not reshape the overall diversity and global composition of intestinal microbiota in Amur sturgeon.

Taxonomic community profiles were further analyzed at the phylum and genus levels using the top-10 most-abundant taxa. At the phylum level, Proteobacteria exhibited the highest relative abundance in the CK group (40.09% of all valid sequences). In contrast, Firmicutes (including Firmicutes_A, Firmicutes_D, and Firmicutes_C) showed the highest relative abundance in ML-treated groups, with relative abundances of 51.44%, 46.18%, and 48.98% in the ML2, ML4, and ML6 groups, respectively. Compared with the CK group, all ML supplementation groups displayed decreased relative abundance of Actinobacteriota, while the relative abundance of Bacteroidota increased progressively with increasing dietary ML inclusion levels (Figure 3C). These phylum-level observations are presented as numerical abundance patterns; no statistical comparisons were performed for individual phylum-level relative abundances.

At the genus level, *Clostridium_P* and *Dwaynesavagella* displayed the most pronounced numerical shifts in relative abundance in response to ML intervention. Specifically, *Clostridium_P* showed a marked numerical increase in the ML4 group, whereas *Dwaynesavagella* exhibited the highest relative abundance in the ML6 group. Multiple potential probiotic genera, such as *Lactococcus_A* and *Bacillus_P*, also showed notably higher relative abundances in ML-supplemented groups compared with the CK group (Figure 3D). As with the phylum-level analysis, these genus-level relative-abundance shifts are described as numerical trends and were not subjected to formal statistical testing.

LEfSe analysis was performed to screen differential microbial biomarkers among different dietary groups. Three unique differential genera, including *Cypionkella*, *Escherichia*, and *Clostridium_T*, were identified as characteristic biomarkers of the CK group. Order Bacillales_B and genus *Lysinibacillus* served as specific biomarkers for the ML2 group. The ML6 group possessed the richest differential taxa, including order Rhodobacterales, family Rhodobacteraceae, and genus *Priestia*. Notably, *Saccharofermentans* was verified as a unique biomarker of the ML6 group, with significant differential abundance at the order, family, and genus taxonomic levels (Figure 3E).

### 3.5. Metabolite Distribution and KEGG Pathway Enrichment Analysis of A. schrenckii

PCA was carried out to assess the repeatability of untargeted metabolomics data. The tight clustering of all QC samples confirmed stable instrumental operation and favorable analytical reproducibility. Metabolic features with a relative standard deviation (RSD) > 30% among QC samples were removed prior to downstream analyses. OPLS-DA models were established to distinguish metabolic profiles between groups. Permutation testing excluded model overfitting and ensured the reliability of the OPLS-DA outputs.

**Figure 4:**
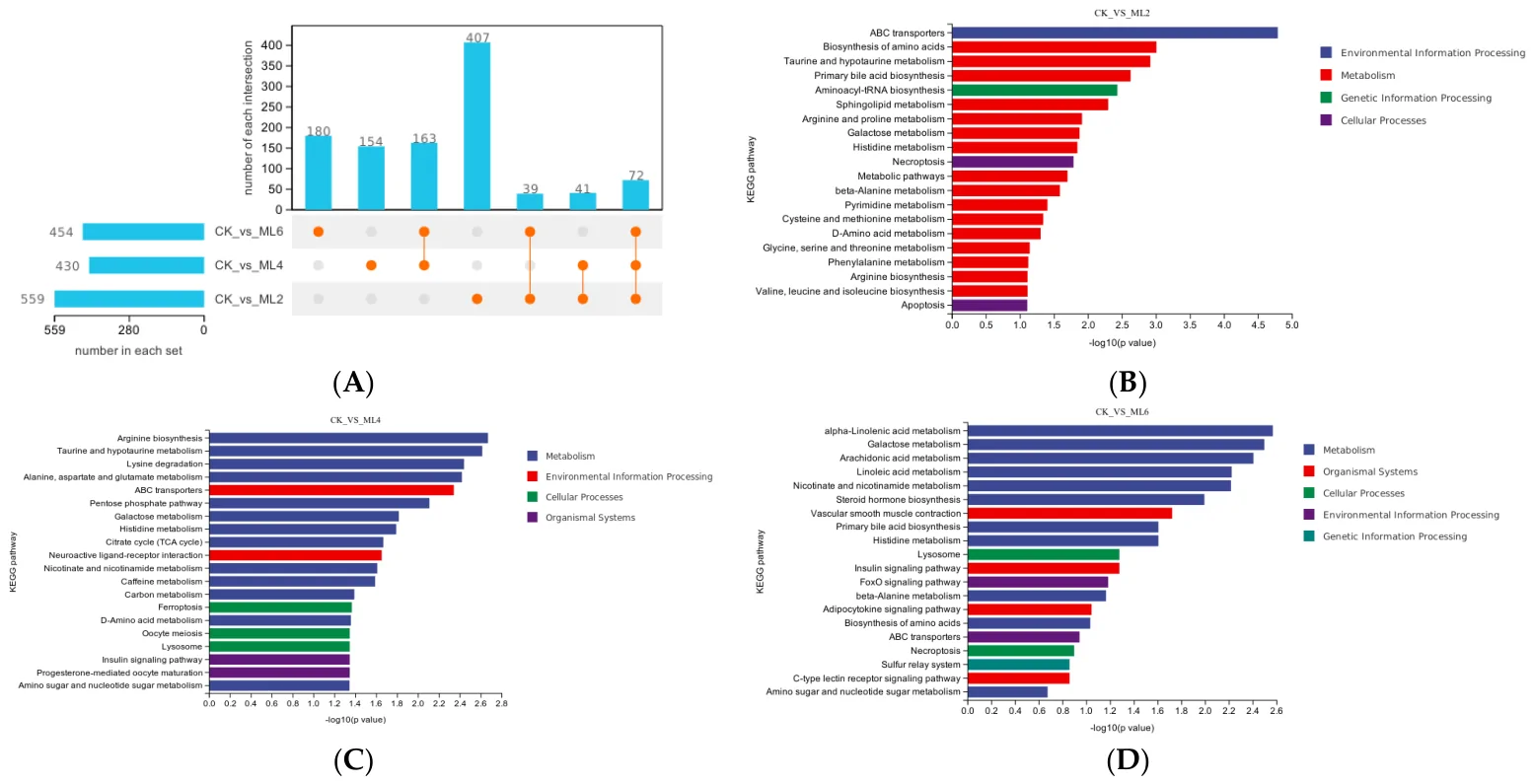
Differential metabolite distribution and KEGG pathway enrichment analysis (*n* = 6). (**A**) UpSet plot showing the distribution of shared and unique differential metabolites among comparison groups. (**B**–**D**) KEGG pathway enrichment analysis of differential metabolites derived from the comparisons of CK vs. ML2 (**B**), CK vs. ML4 (**C**), and CK vs. ML6 (**D**), respectively. The *x*-axis represents- log10 (*p*-value) and the *y*-axis represents KEGG Pathway.

Compared with the CK group, a total of 72 differential metabolites were identified across all mulberry leaf treatment groups (Figure 4A). These metabolites were assigned to diverse chemical classes: 1 alkaloid and derivative, 4 benzenoids, 13 lipids and lipid-like molecules, 12 organic acids and derivatives, 5 organic oxygen compounds, 10 organoheterocyclic compounds, and 3 phenylpropanoids and polyketides, alongside other miscellaneous metabolites. Filtered by thresholds of VIP ≥ 1.7 and |FC| ≥ 3, the top-12 prominent altered metabolites consistently up-regulated in mulberry-leaf supplemented groups were epoxyartemisia ketone, trifolin, flavonoid glycoside-1 [2-(3,4-dihydroxyphenyl)-5-hydroxy-4-oxo-4H-chromen-7-yl 6-O-(6-deoxy-alpha-L-mannopyranosyl)-beta-D-glucopyranoside], putative (3-hydroxyhexadecanoyl)glycine (commendamide), salmeterol, herbimycin A, 1-(7H-purin-6-yl)-N-[3-(trifluoromethyl)phenyl]-4-piperidinecarboxamide, harderoporphyrin, pheophorbide A, ceramide (d18:1/16:0), coproporphyrin III, and flavonoid glycoside-2 [5,7-dihydroxy-2-(4-hydroxyphenyl)-4-oxo-4H-chromen-3-yl 6-O-(6-deoxyhexopyranosyl)hexopyranoside]. Among these compounds, 32 metabolites were continuously upregulated, whereas 35 were persistently downregulated in the ML2, ML4 and ML6 groups.

KEGG enrichment analysis was performed for each ML group versus CK to characterize changes in metabolic pathways. In the ML2 group, ABC transporters represented the most significantly enriched pathway, and most enriched pathways were involved in basal metabolism, including biosynthesis of amino acids, taurine and hypotaurine metabolism, and primary bile acid biosynthesis (Figure 4B). For the ML4 group, arginine biosynthesis and taurine and hypotaurine metabolism ranked as the top enriched pathways. Two core carbon metabolism pathways—the tricarboxylic acid (TCA) cycle and pentose phosphate pathway—as well as the lysosome pathway associated with antioxidant responses, were newly enriched (Figure 4C). In the ML6 group, pathways participating in unsaturated fatty acid metabolism were significantly enriched, including α-linolenic acid metabolism, galactose metabolism and arachidonic acid metabolism. Meanwhile, more antioxidant- and immune-related pathways were enriched in ML6, such as lysosome, FoxO signaling pathway and C-type lectin receptor signaling pathway (Figure 4D).

### 3.6. Gene Expression and Functional Enrichment Analyses of A. schrenckii

Raw RNA-seq reads were subjected to quality trimming. The Q30 values of all samples were higher than 97.40%, verifying high-quality sequencing data. Clean reads were mapped to the reference genome, and gene expression abundance was quantified for the screening of DEGs.

**Figure 5:**
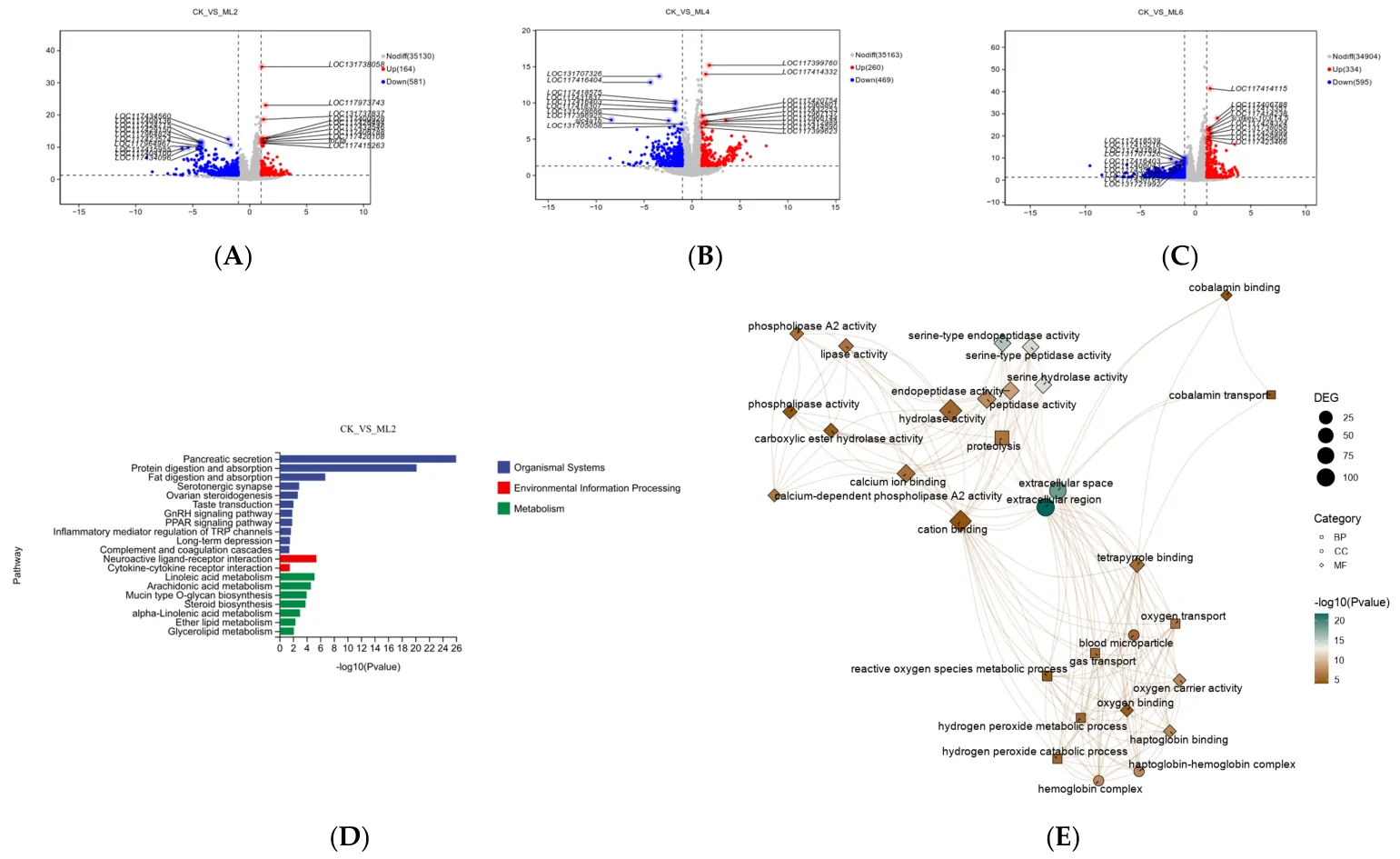

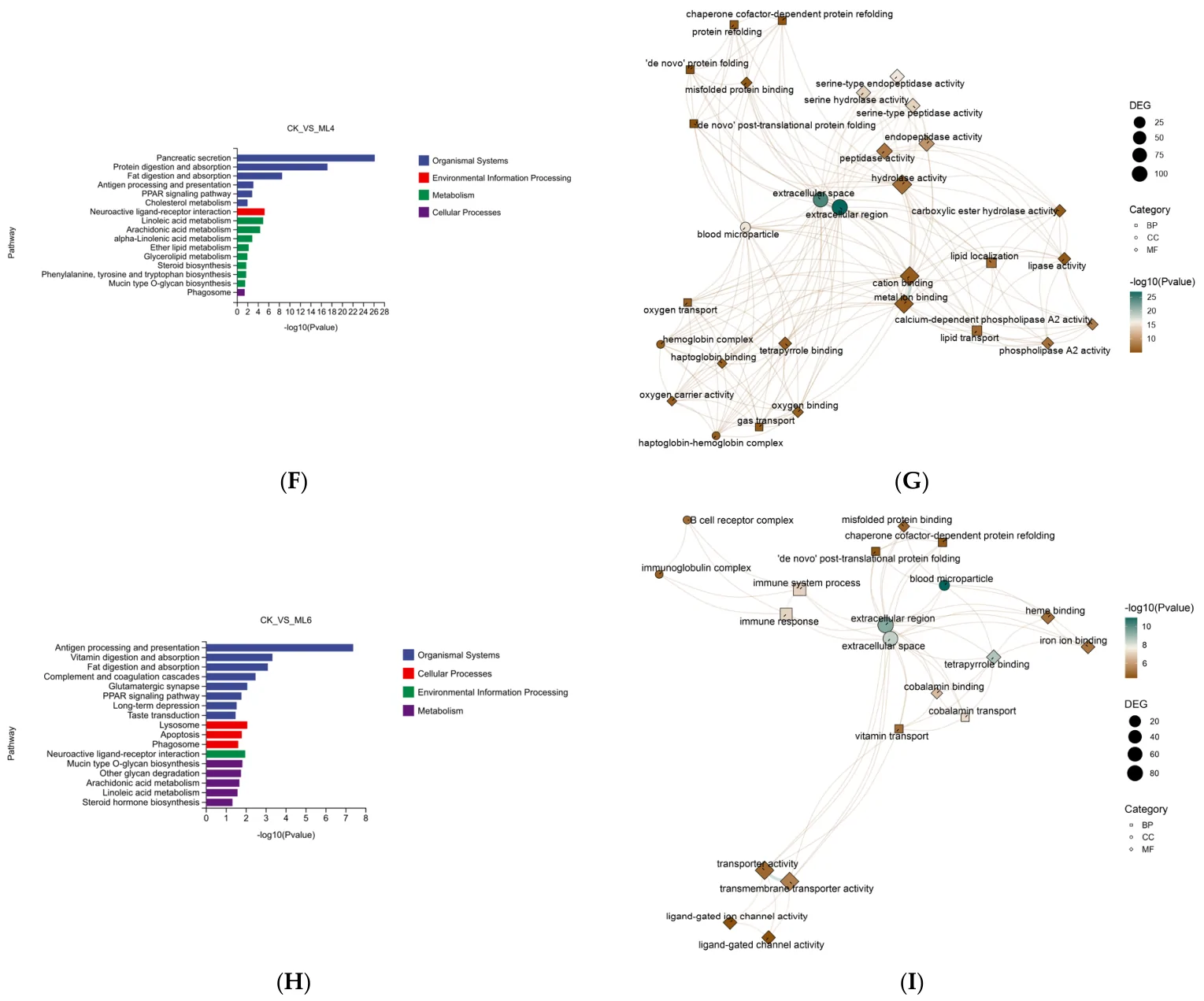
Differential gene expression and functional enrichment analyses of the three comparison groups (*n* = 6). (**A**–**C**) Volcano plots of DEGs in the CK vs. ML2 (**A**), CK vs. ML4 (**B**), and CK vs. ML6 (**C**), respectively. The horizontal axis represents log_2_(fold change), and the vertical axis denotes −og_10_(*p*-value). The two vertical dashed lines indicate the threshold of fold change, while the horizontal dashed line represents the significance threshold. Genes are colored to show significantly upregulated, significantly downregulated, and non-significantly differentially expressed genes. (**D**–**F**) KEGG pathway enrichment bar charts for the CK vs. ML2 (**D**), CK vs. ML4 (**E**), and CK vs. ML6 (**F**), respectively. The vertical axis lists enriched KEGG pathways, and the horizontal axis corresponds to −log_10_(*p*-value) of pathway enrichment. (**G**–**I**) GO enrichment circle plots for the CK vs. ML2 (**G**), CK vs. ML4 (**H**), and CK vs. ML6 (**I**), respectively. Each circle stands for one GO term; the size of the circle indicates the number of enriched DEGs within the corresponding GO term, and the color gradient reflects the enrichment significance. The thickness of connecting lines represents the number of shared DEGs between paired GO terms, where thicker lines indicate more overlapping DEGs.

Compared with the CK group, 164, 260 and 334 genes were significantly upregulated in the ML2, ML4 and ML6 groups, whereas 581, 469 and 595 genes were significantly downregulated, respectively (Figure 5A–C). KEGG enrichment analysis was conducted on DEGs from each pairwise comparison. Pancreatic secretion and protein digestion and absorption were the most significantly enriched pathways in both ML2 and ML4 groups, together with multiple metabolic pathways such as linoleic acid metabolism and arachidonic acid metabolism. The cytokine–cytokine receptor interaction pathway was specifically enriched in ML2 (Figure 5D). Immune-associated pathways, including antigen processing and presentation and phagosome, were significantly enriched in ML4 (Figure 5E). The ML6 group displayed unique pathway enrichment profiles, with antigen processing and presentation as the top enriched pathway. Several pathways involved in cellular homeostasis and innate immunity, such as lysosome, apoptosis and phagosome, were enriched in ML6 (Figure 5F).

GO enrichment network analysis was performed based on DEGs obtained from the CK vs. ML2, CK vs. ML4 and CK vs. ML6 comparisons. Extracellular region and extracellular space were recognized as core shared CC hub terms across all mulberry leaf treatments. GO terms from the CK vs. ML2 comparison were grouped into three functional modules. The upper-left cluster was associated with nutrient digestion and hydrolysis, covering serine-type endopeptidase activity, serine-type peptidase activity and carboxylic ester hydrolase activity. The lower-right cluster contained terms related to oxygen transport and antioxidant homeostasis, including hemoglobin complex, reactive oxygen species metabolic process and hydrogen peroxide catabolic process. A separate small cluster was involved in vitamin transport and metabolism, represented by cobalamin binding and cobalamin transport (Figure 5G). Four functional clusters were observed for CK vs. ML4. Similar to ML2, the digestion-hydrolysis and oxygen transport-antioxidant modules remained enriched with increased enrichment strength. An additional cluster associated with protein folding and misfolded protein repair emerged in the upper-left region, characterized by misfolded protein binding and de novo protein folding. The central-right cluster corresponded to cation/metal ion binding and lipid transport (Figure 5H). ML6 exhibited substantially reorganized GO enrichment patterns consisting of four major modules. Immune response-related terms (B cell receptor complex, immunoglobulin complex, immune system process) formed the predominant upper-left cluster. The protein folding module detected in ML4 was retained. Glycoside catabolism and metabolism dominated the carbohydrate metabolism module in the upper-right region. A series of transporter activity and ion transport terms constituted the transmembrane transport module in the lower-left region (Figure 5I).

### 3.7. Integrated Analysis of Gut Microbiota and Metabolome

Spearman rank correlation heatmaps were used to visualize pairwise associations between genus-level differential intestinal bacteria and differential metabolites under graded dietary mulberry leaf (ML) supplementation. Distinct correlation patterns were identified among the ML2, ML4 and ML6 treatment groups.

**Figure 6:**
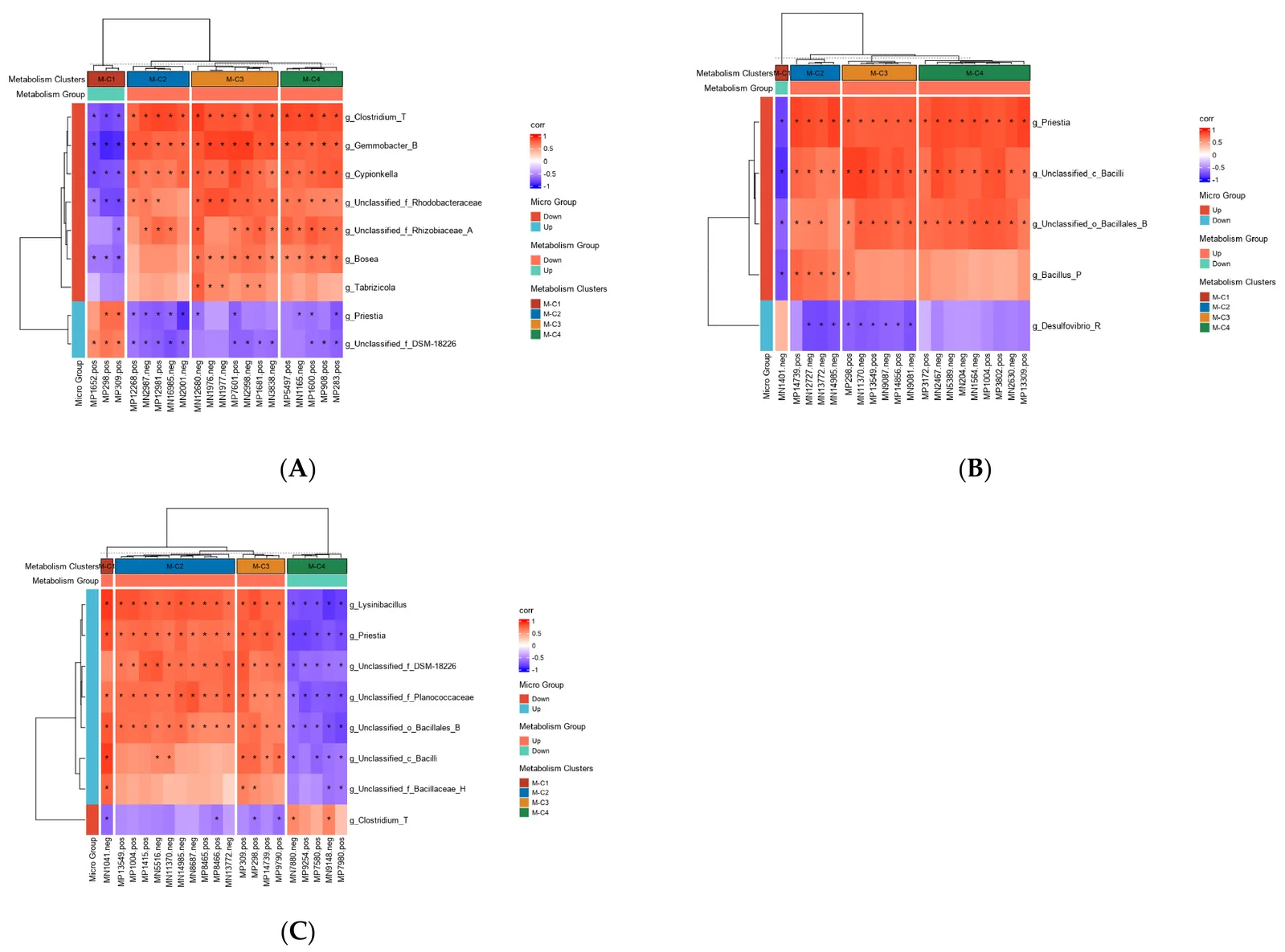
Correlation heatmap of differential gut microbial genera and metabolites under graded ML supplementation (*n* = 6). (**A**) ML2 vs. CK; (**B**) ML4 vs. CK; (**C**) ML6 vs. CK. The horizontal axis represents differential gut microbial genera at the genus level, and the vertical axis represents differential metabolites. Red color indicates significant positive correlations, while blue-purple color indicates significant negative correlations. Asterisks denote statistically significant correlations (*p* < 0.05).

In the low-dose ML2 group, ML-enriched genera (*Priestia*, unclassified_f_DSM-18226) exhibited significant positive correlations with metabolites in cluster M-C1 and significant negative correlations with metabolites belonging to clusters M-C2, M-C3 and M-C4. In contrast, genera depleted in ML2 (which were abundant in the CK group), including *Clostridium_T*, *Gemmobacter_B*, *Cypionkella*, unclassified_f_Rhodobacteraceae, unclassified_f_Rhizobiaceae_A, *Bosea* and *Tabrizicola*, were positively correlated with metabolites in M-C2/M-C3/M-C4 and negatively correlated with metabolites in M-C1 (Figure 6A).

In the medium-dose ML4 group, ML-upregulated genera (*Priestia*, unclassified_c_Bacilli, unclassified_o_Bacillales_B, *Bacillus_P*) showed extensive positive correlations with metabolites from M-C2 to M-C4 and negative correlations with downregulated metabolites. The single genus depleted in ML4 (*Desulfovibrio_R*) presented an opposite correlation profile: negatively associated with upregulated metabolites and positively associated with downregulated metabolites (Figure 6B).

In the high-dose ML6 group, all ML-enriched genera displayed significant positive correlations with metabolites in M-C1, M-C2 and M-C3, together with negative correlations with metabolites in M-C4. The predominant CK-enriched genus *Clostridium_T* exhibited inverse relationships: negative correlations with M-C1/M-C2/M-C3 and a positive correlation with M-C4 (Figure 6C).

### 3.8. Integrated Analysis of Intestinal Transcriptome and Metabolome

Pearson correlation heatmaps were generated to characterize pairwise associations between DEGs and differential metabolites under graded dietary mulberry leaf (ML) supplementation. Distinct gene–metabolite correlation patterns were observed among the ML2, ML4, and ML6 treatment groups.

**Figure 7:**
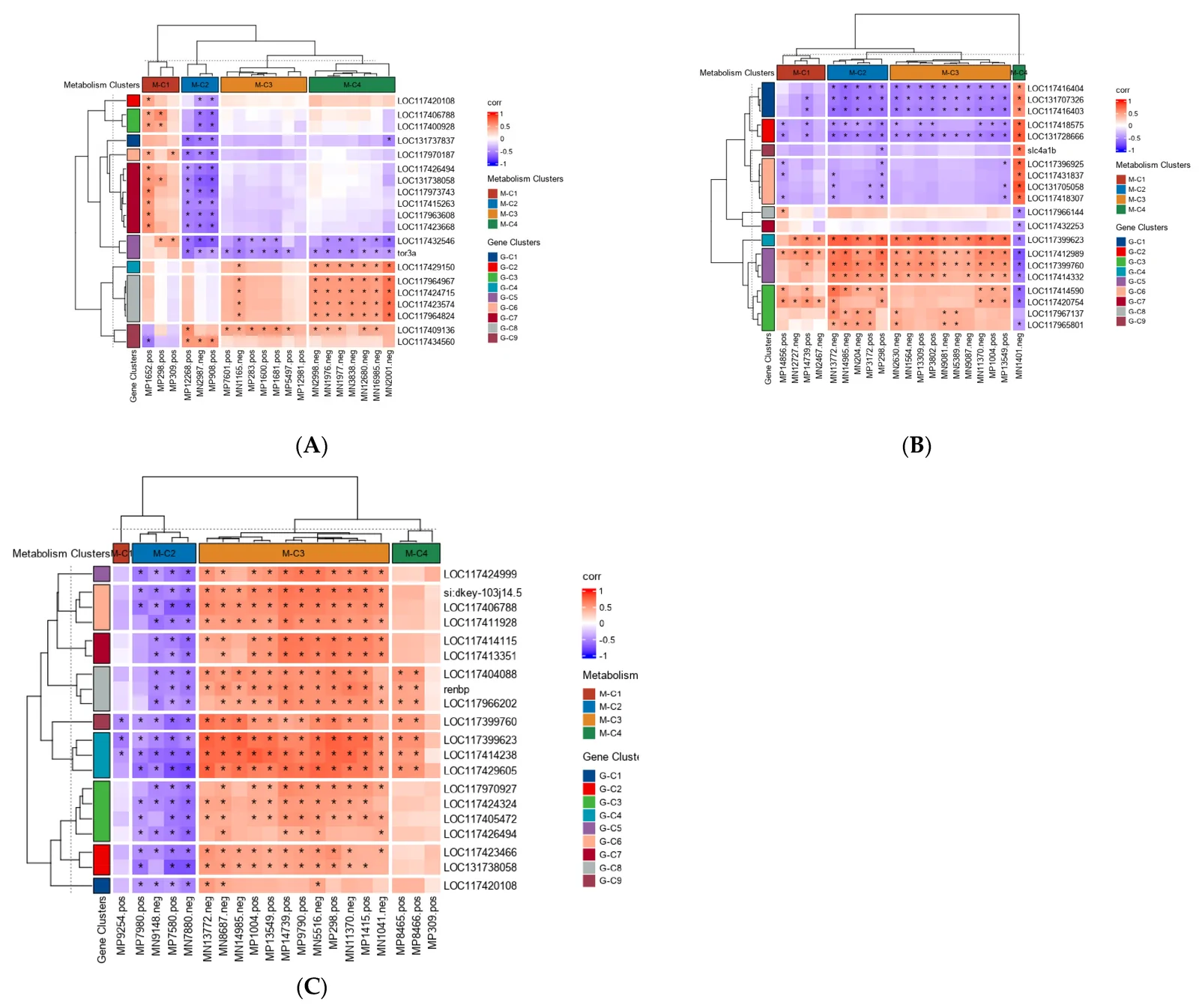
Pearson correlation heatmap of differentially expressed genes and differential metabolites under graded ML supplementation (*n* = 6). (**A**) ML2 vs. CK; (**B**) ML4 vs. CK; (**C**) ML6 vs. CK. The horizontal axis represents gene names of DEGs, and the vertical axis represents differential metabolites. Red blocks indicate significant positive correlations, while blue-purple blocks indicate significant negative correlations. Asterisks mark statistically significant correlations at *p* < 0.05.

In the low-dose ML2 group, gene clusters G-C1 to G-C3 and G-C5 to G-C7 were significantly negatively correlated with metabolite cluster M-C2. In addition, the G-C5 cluster exhibited significant negative correlations with M-C3 and M-C4. By contrast, gene clusters G-C4 and G-C8 showed significant positive correlations with M-C4 (Figure 7A). The medium-dose ML4 group presented unique correlation characteristics. Specifically, G-C1 and G-C2 were negatively correlated with M-C2 and M-C3 but positively correlated with M-C4, while G-C4 and G-C5 were positively associated with M-C2 and M-C3 and negatively associated with M-C4 (Figure 7B). The high-dose ML6 group exhibited consistent overall correlation trends across all detected clusters: all gene clusters were significantly negatively correlated with M-C2 and significantly positively correlated with M-C3 (Figure 7C).

### 3.9. Quantitative Real-Time PCR Validation of Differentially Expressed Genes

**Figure 8:**
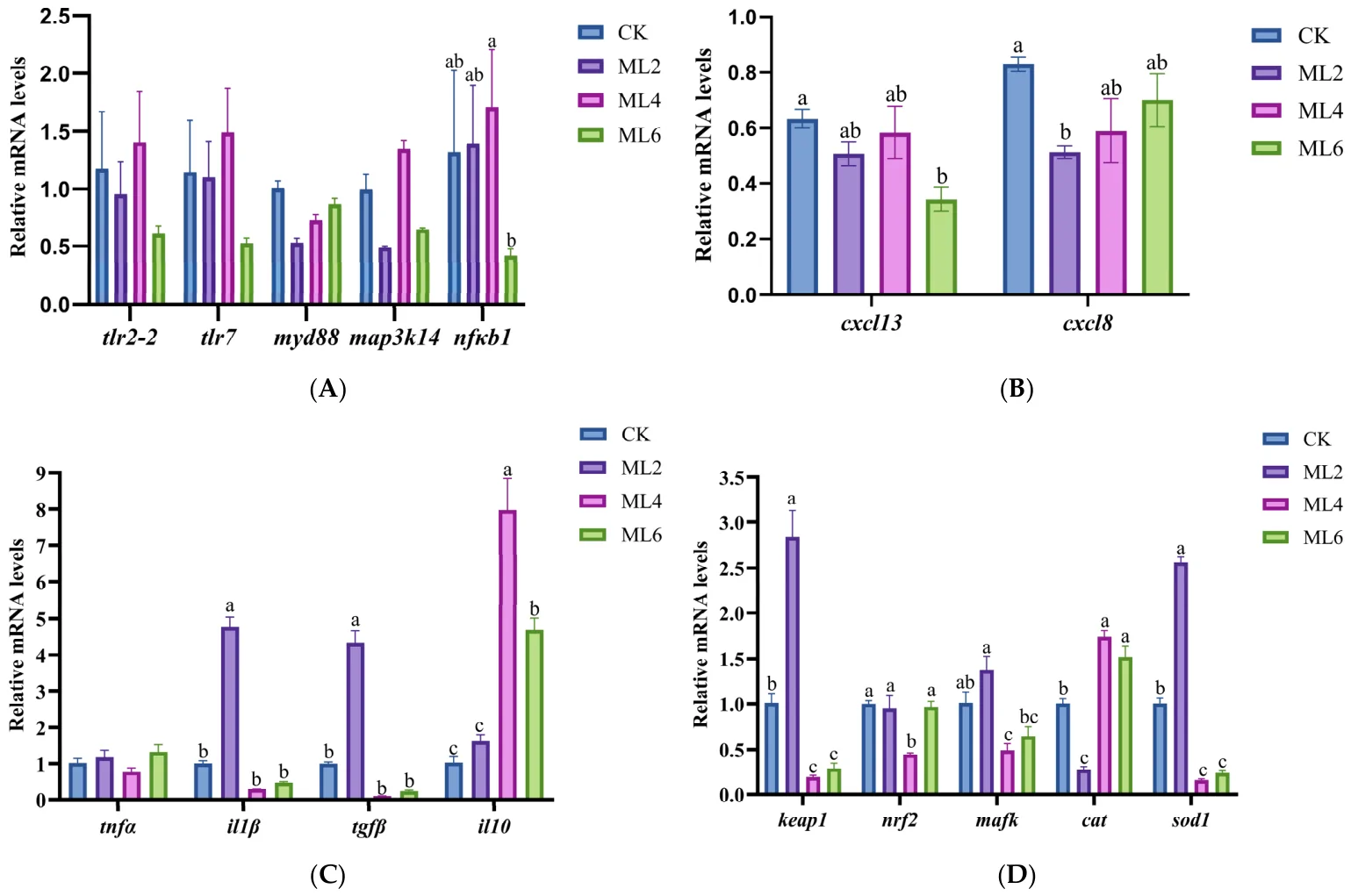
Relative mRNA expression of immune, inflammatory and antioxidant-related genes across experimental groups (*n* = 3). (**A**) TLR-NF-κB pathway genes; (**B**) Chemokine genes; (**C**) inflammatory cytokine genes; (**D**) antioxidant-related genes. Data are mean ± SEM. Different lowercase letters indicate significant differences (*p* < 0.05).

The mRNA expression levels of upstream genes involved in the TLR-NF-κB signaling pathway, including *tlr2-2*, *tlr7*, *myd88*, and *map3k14*, showed no significant differences among all mulberry leaf treatment groups compared with the CK group. The transcript abundance of *nfkb1* was significantly higher in the ML4 group than in the ML6 group (*p* < 0.05; Figure 8A). For chemokine genes, *cxcl13* expression was significantly downregulated in the ML6 group, while *cxcl8* expression was markedly decreased in the ML2 group relative to CK (*p* < 0.05; Figure 8B).

In terms of inflammatory cytokine genes, no significant intergroup differences were observed in *tnfα* expression. The pro-inflammatory genes *il1β* and *tgfβ* were significantly upregulated in the ML2 group compared with the CK, ML4, and ML6 groups. The anti-inflammatory gene *il10* exhibited significantly higher mRNA abundance in the ML4 and ML6 groups than in the CK and ML2 groups (*p* < 0.05; Figure 8C).

For antioxidant-related genes, *keap1* expression was significantly higher in the CK and ML2 groups than in the ML4 and ML6 groups. The *sod1* expression level peaked in the ML2 group (*p* < 0.05). Both *nrf2* and *mafk* expression were significantly lower in the ML4 group than in the CK group (*p* < 0.05). Additionally, *cat* expression reached its minimum value in the ML2 group, and the CK group showed significantly lower *cat* transcript abundance compared with the ML4 and ML6 groups (*p* < 0.05) (Figure 8D).

## 4. Discussion

Consistent with growth performance and intestinal digestive enzyme data across all groups, graded dietary mulberry leaf inclusion exerted no adverse effects on somatic growth and intestinal digestive capacity. These findings exclude retarded growth and digestive dysfunction as confounding factors, indicating that the observed changes in intestinal antioxidant capacity, inflammatory response and intestinal barrier function are likely driven by mulberry leaf-mediated regulation of intestinal chemical and immune barrier homeostasis.

### 4.1. Graded Mulberry Leaf Supplementation Selectively Enriches Beneficial Intestinal Functional Microbiota

The intestinal microbiota plays a pivotal role in regulating host metabolism, nutrient absorption, and immune function in aquatic species [21,22]. In the present study, graded mulberry leaf supplementation did not alter the overall intestinal microbial community structure of Amur sturgeon, and no significant intergroup differences were detected in alpha- and beta-diversity. These results suggest that dietary mulberry leaf intervention does not disrupt intestinal microbial homeostasis, reflecting favorable ecological safety for the gut microbiota.

Notably, stable overall microbial diversity and community structure do not exclude subtle but biologically meaningful alterations in individual taxonomic taxa. Taxonomic analysis revealed that dietary mulberry leaf reduced the relative abundance of unclassified microbial taxa (Others). The intestinal microbial profile shifted from a pattern characterized by high diversity and low dominance toward targeted enrichment of functionally specific genera. Collectively, mulberry leaf facilitates directional regulation of intestinal microbiota, enriching beneficial commensal bacteria while sustaining the stability of the core microbial community.

*Cypionkella* and *Escherichia*, which were enriched in the CK group, belong to the phylum Proteobacteria. *Escherichia* represents a common opportunistic pathogen in aquatic animals [23], and its predominance in control fish may indicate a higher load of potentially harmful bacteria under the basal dietary condition. *Clostridium_T*, an anaerobic taxon capable of producing short-chain fatty acids (SCFAs) that suppress opportunistic pathogens and maintain intestinal homeostasis [24], was also enriched in the CK group. This co-occurrence suggests that *Clostridium_T* may act as a core genus sustaining basal intestinal homeostasis in control sturgeon, possibly via a compensatory mechanism to counterbalance the potential pathogenic pressure exerted by *Escherichia*. In other words, an equilibrium between beneficial and opportunistic bacterial taxa was maintained in the intestinal microbiota of CK fish. The ML2 group exhibited enrichment of *Bacillales_B* and *Lysinibacillus* (phylum Firmicutes). Species within the genus *Bacillus* are widely applied probiotics in aquaculture, as they optimize intestinal microecology and improve stress tolerance [25]. *Lysinibacillus* has been reported to exert antibacterial, antioxidant and anti-stress effects, contributing to the maintenance of intestinal health [26,27]. The ML6 group displayed the most prominent enrichment of differential microbial biomarkers across the order, family and genus taxonomic levels. Rhodobacterales and Rhodobacteraceae harbour abundant genes associated with polyhydroxybutyrate metabolism, which facilitate organic matter degradation to supply energy and support fish growth [28,29]. *Priestia*, a beneficial genus closely related to *Bacillus*, improves intestinal digestive efficiency and enhances host immunity and anti-stress capacity [30]. Taxa including Saccharofermentanales, Saccharofermentanaceae and *Saccharofermentans* are carbohydrate-degrading anaerobes. These microorganisms degrade dietary polysaccharides to promote nutrient utilization and stabilize intestinal microecology via the secretion of short-chain fatty acids [31].

In summary, graded mulberry leaf supplementation remodels the intestinal microecosystem of Amur sturgeon in a dose-dependent manner. Mulberry leaf promotes the enrichment of multiple beneficial microbes and reduces potentially pathogenic taxa without disrupting the overall balance of the intestinal microbial community.

### 4.2. Dose-Dependent Intestinal Metabolic Remodeling with Multi-Functional Gradient Regulation

Intestinal metabolic profiles reflect integrated host–microbe regulation of nutrient digestion, metabolism and immune homeostasis. Elucidating metabolic shifts triggered by dietary functional plant ingredients helps uncover their physiological mechanisms and graded regulatory characteristics [32,33]. Our metabolomic results demonstrated that graded dietary mulberry leaf supplementation induced distinct intestinal metabolic signatures, supporting a layered functional regulatory pattern.

Metabolic modulation in the ML2 group was dominated by improved basal nutrient metabolism, accompanied by significant enrichment of ABC transporters, biosynthesis of amino acids, taurine and hypotaurine metabolism, and primary bile acid biosynthesis. ABC transporters form an energy-dependent transmembrane transport system broadly involved in nutrient uptake, molecular transport, immune regulation and disease progression, acting as key mediators of cellular homeostasis and environmental adaptation [34,35]. In aquatic organisms, taurine and bile acids generally enhance antioxidant capacity and immunity. Taurine scavenges excess free radicals to alleviate oxidative damage and promotes immune cell activation, thereby improving systemic immune competence [36,37]. Bile acids increase SOD and T-AOC activities while decreasing MDA levels to elevate whole-body antioxidant capacity [38,39]. These biological functions align well with the improved serum antioxidant indicators observed in the ML2 group. Accordingly, low-dose mulberry leaf supplementation facilitates growth and metabolic stability by enhancing intestinal nutrient transport and basal metabolism.

In the ML4 group, metabolic regulation transitioned from basal nutrient metabolism toward stress tolerance and immune homeostasis; arginine biosynthesis and taurine metabolism represented the most significantly enriched pathways. As an indispensable amino acid for fish, arginine participates in non-specific immune responses and antioxidant reactions and improves stress and disease resistance [40]. This metabolic feature is consistent with the elevated serum IgM and SOD as well as reduced MDA detected in ML4. Furthermore, the lysosome pathway was enriched. This pathway mediates substrate degradation, autophagy, immune defence and tissue homeostasis, and eliminates damaged cells to relieve intestinal stress injury [41]. Therefore, medium-dose mulberry leaf supplementation maintains efficient nutrient utilization while initiating antioxidant- and immune-related metabolic cascades, coordinating growth performance and stress resistance.

Metabolic remodelling in the ML6 group focused on lipid metabolism reprogramming and the maintenance of intestinal immune homeostasis, with marked enrichment in pathways related to unsaturated fatty acids, including α-linolenic acid, arachidonic acid and linoleic acid metabolism. This metabolic pattern matches the function of *Saccharofermentans*, the ML6-specific biomarker genus that generates short-chain fatty acids to modulate lipid metabolism. Polyunsaturated fatty acids are essential membrane components involved in signal transduction, lipid turnover and immune modulation, and they benefit intestinal health and immunity in fish [42,43,44]. These functions correspond to the changes in serum immune parameters observed in the present trial. In addition to sustained enrichment of basal metabolic pathways (ABC transporters, biosynthesis of amino acids, bile acid metabolism), the ML6 group exhibited enrichment of the FoxO signalling pathway, which is linked to antioxidant defence and immune regulation [45]. Overall, high-dose mulberry leaf establishes a multilayered metabolic network supporting nutrient utilization, redox balance and immune enhancement.

In conclusion, mulberry leaf exerts dose-specific regulatory effects on intestinal metabolism in Amur sturgeon. Low doses optimize nutrient metabolism to favour growth; medium doses prioritize antioxidation and anti-stress capacity; high doses remodel lipid metabolism to strengthen intestinal immune homeostasis. These divergent metabolic phenotypes confirm that mulberry leaf shapes distinct intestinal steady-state features through dosage adjustment.

### 4.3. Dose-Dependent Improvement of Intestinal Chemical Barrier, Oxidative Homeostasis and Mucosal Permeability

The intestinal chemical barrier consists of diverse endogenous compounds that block the invasion of exogenous pathogens. Glycosylated mucins synthesized and secreted by goblet cells are critical for resisting external stimuli and pathogenic infection [46]. Transcriptomic KEGG enrichment revealed that the mucin type O-glycan biosynthesis pathway was enriched in all three mulberry leaf groups, with the highest enrichment observed in ML2. Histological data further confirmed significantly higher goblet cell abundance in ML2 relative to CK. Activation of mucin biosynthesis promotes goblet cell proliferation and differentiation; abundant secreted mucins coat the intestinal epithelium and form a dense mucus-based chemical barrier [47]. RT-qPCR detected elevated transcription of *keap1* and *sod1* in ML2, consistent with increased serum SOD and reduced MDA. These observations support the notion that low-dose mulberry leaf maintains the integrity of the intestinal chemical barrier. By eliminating excess intestinal reactive oxygen species and inhibiting lipid peroxidation, oxidative degradation of the mucus layer is restrained, preserving the structural integrity of the mucus barrier [48].

The antioxidant gene *cat* was significantly upregulated in ML4. The PPAR-mediated lipid metabolism pathway suppresses oxidative stress cascades. Consistently, serum T-AOC and SOD reached peak values in ML4, indicating enhanced systemic antioxidant capacity. These alterations further stabilize the intestinal chemical barrier and create a favourable microenvironment for epithelial barrier repair [49]. Notably, transcript levels of *nrf2* and *mafk* were lower in ML4 than in CK. This finding suggests that mulberry leaf-induced antioxidant enhancement may operate independently of the canonical Nrf2–Keap1 axis [50]. Antioxidant regulation exhibited transcriptional reprogramming among different effector genes: *sod1* expression decreased, whereas transcription of *cat* increased as a potential compensatory mechanism.

Intestinal DAO activity is a well-established biomarker of intestinal mucosal damage and epithelial permeability [51]. DAO activity declined significantly in ML4 and ML6, indicating that mulberry leaf reshapes intestinal defence: the simple superficial barrier dominated by mucus transitions toward dual protection integrating a mucus-associated antioxidant microenvironment and intact epithelial barrier. This dual defence limits the translocation of intestinal endotoxins and harmful metabolites into systemic circulation and the liver, alleviating intestinal-derived metabolic injury [52]. Although goblet cell counts in ML4 and ML6 returned to the levels of the CK group, early establishment of the mucus barrier combined with optimized antioxidant status remodelled intestinal protection. The barrier regulatory mode shifted from compensatory overproduction of mucus toward sustained long-term protection dependent on stable redox homeostasis [53].

### 4.4. Dose-Dependent Sequential Modulation of the Intestinal Immune Barrier upon Graded Dietary Mulberry Leaf Supplementation

The intestinal immune barrier, composed of gut-associated lymphoid tissues, immune cells and secreted immunoglobulins, constitutes the core defensive component of the entire intestinal barrier system.

The ML2 group displayed moderate innate immune activation to initiate protective mucosal immunity. Transcriptomic KEGG enrichment revealed prominent enrichment of the cytokine–cytokine receptor interaction pathway, a major downstream cascade of TLR/NF-κB innate immune signalling governing intestinal inflammatory responses [54]. RT-qPCR showed no significant transcriptional variation in upstream *tlr* family genes across groups. It is speculated that beneficial intestinal commensals modulate NF-κB activity at the post-translational level, leading to significant upregulation of *il1β* and *tgfβ* [55]. Moderate secretion of *il1β* activates innate immune cells including intestinal epithelial cells and macrophages, while *tgfβ* maintains immune tolerance to avoid pathological inflammation triggered by excessive innate immune overactivation [56]. Collectively, low-dose mulberry leaf reinforces mucosal innate immunity via homeostatic protective regulation.

In ML4, *nfkb1*-driven upregulation of *cxcl13* facilitated intestinal B-cell homing, linking innate immunity and adaptive immunity. Transcriptomic data confirmed enrichment of antigen processing and presentation, an essential prerequisite for the activation of intestinal adaptive immunity [57]. RT-qPCR verified peak transcription of *nfkb1* in ML4. This transcription factor directly promotes *cxcl13* expression; CXCL13 recruits circulating B lymphocytes to colonize the intestinal lamina propria and establishes the cellular foundation for mucosal humoral immunity [58,59]. Following the initiation of B-cell homing, NF-κB signalling simultaneously induced high transcription of *il10*. On the one hand, *il10* provides negative feedback to restrict excessive NF-κB signalling and block pro-inflammatory cascades, preventing intestinal inflammatory injury induced by immune imbalance [60]. On the other hand, *il10* drives macrophages and dendritic cells toward anti-inflammatory phenotypes [61,62]. Combined with *cat*-dependent antioxidant defence, these pathways cooperatively restore intestinal epithelial tight junctions, accompanied by reduced DAO activity. The synergistic protective effects exerted by immune and chemical barriers on the intestinal mucosa reached a maximum in the ML4 group.

The ML6 group establishes long-term adaptive immune homeostasis relying on B cell receptor signalling. GO enrichment identified specific enrichment of the B cell receptor complex, immunoglobulin complex and general immune response modules in ML6. This demonstrates that CXCL13-recruited B lymphocytes complete mucosal colonization and mature differentiation to build durable adaptive immune defence [63]. Briefly, high *nfkb1* expression in ML4 transiently induced *cxcl13* transcription to accomplish B-cell recruitment. As dietary mulberry leaf dosage increased to the ML6 level, transcription of *nfkb1* and *cxcl13* decreased sharply to avoid excessive lymphocyte infiltration within the lamina propria. Colonized mature B cells no longer rely on continuous CXCL13-mediated chemotaxis; instead, they utilize B cell receptor signalling to sustain long-term intestinal immune tolerance [64]. Intestinal resident B cells continuously express B cell receptors, recognize antigens derived from intestinal commensal bacteria to maintain immune tolerance homeostasis, and further differentiate into plasma cells for immunoglobulin synthesis and secretion [65]. Elevated serum IgM and C3 across all mulberry leaf groups confirmed that mucosal B-cell activation triggered systemic humoral immune responses. Regulatory B cells serve as the major cellular source of persistently high *il10* secretion, limiting intestinal inflammation and constructing a regulatory mucosal immune barrier [66]. Transcriptomic enrichment of lysosome and apoptosis pathways enables timely clearance of senescent and oxidatively damaged epithelial cells [67] and supports intestinal stem cell proliferation. Consistently, crypt depth increased significantly in ML6, accelerating intestinal epithelial turnover and strengthening the physical barrier, thereby forming coordinated multilayered defence together with immune and chemical barriers. As a biomarker of intestinal epithelial repair and mucosal homeostasis [68], ALP activity increased significantly in ML6, reflecting robust epithelial proliferation, repair and barrier remodelling under high-dose mulberry leaf supplementation. Sustained reduction in intestinal permeability alleviates hepatic endotoxin translocation. Combined with PPAR-mediated hepatic lipid metabolic homeostasis, the hepatosomatic index declined significantly in ML6, achieving multi-barrier synergistic protection along the gut–liver axis [49,52].

Collectively, transcriptomic and RT-qPCR evidence demonstrates that mulberry leaf remodels intestinal immunity following a three-stage sequential cascade: initial innate immune priming, mucosal B-cell homing, and establishment of long-term tolerogenic immune homeostasis. The complete regulatory axis follows a clear upstream-to-downstream sequence: graded mulberry leaf reshapes intestinal microbiota and intestinal metabolism; improved mucus secretion, redox balance and epithelial integrity stabilize the intestinal chemical barrier; a stabilized chemical barrier enables orderly, staged activation of mucosal immunity. Homeostasis of the chemical barrier and immune remodelling act synergistically to maintain gut–liver immune and chemical homeostasis in Amur sturgeon.

## 5. Limitations

Several limitations should be considered. First, although diets were isonitrogenous and isoenergetic, mulberry leaf inclusion required concomitant adjustments to other dietary components (fish meal, wheat flour, gluten meal), which may have introduced variations in protein source ratios and pellet physical properties. While the significant FCR improvement at ML2 and the overall linear dose–response trend is consistent with an independent effect of mulberry leaf bioactives on feed utilization, the specific contribution of mulberry leaf cannot be fully separated from concomitant changes in other dietary components. Second, juvenile sturgeons of mixed sex were used, as sex cannot be determined externally at this stage, potentially contributing to inter-individual variability. Third, the 10-week trial under controlled conditions may not fully reflect long-term commercial production outcomes. Future studies using sex-matched cohorts, substitution designs with constant fish-meal levels, and purified mulberry leaf extracts would be valuable to confirm these findings.

## 6. Conclusions

Within the scope of the present experiment, graded dietary supplementation with mulberry leaf exerted no negative impacts on the growth performance or intestinal digestive capacity of *A. schrenckii*. Mulberry leaf elicited mild and targeted regulation of intestinal microecology: beneficial functional bacterial taxa were enriched in a dose-dependent fashion, while the overall stability of the intestinal microbial community was preserved. Progressive elevation of dietary mulberry leaf drove stepwise reprogramming of intestinal metabolism, generating dosage-specific metabolic signatures that support sustained growth, stress resistance and immune modulation. Furthermore, mulberry leaf strengthened the intestinal chemical barrier, balanced redox homeostasis and reduced intestinal epithelial permeability. Mechanistically, mulberry leaf remodels intestinal immunity through a sequential three-stage cascade: moderate innate immune priming, recruitment and mucosal colonization of B cells, and the establishment of long-term tolerogenic immune homeostasis. This immune regulation acts synergistically with enhanced chemical barrier function to sustain comprehensive intestinal health. From an applied perspective, a preliminary economic assessment based on feed cost per kilogram of weight gain showed that the 2% mulberry leaf inclusion level had the lowest production cost (14.50 yuan/kg vs. 15.06 yuan/kg for the control), suggesting a potential cost advantage that warrants further validation under commercial conditions.

Collectively, this study uncovers the intricate, dose-dependent regulatory network through which mulberry leaf maintains intestinal chemical–immune barrier homeostasis, as revealed by multi-omics integration. These findings advance our mechanistic understanding of how plant-derived bioactive compounds modulate mucosal innate and adaptive immunity in sturgeon.

## Data Availability

Dataset available on request from the authors.

## Supplementary Materials

The following supporting information can be downloaded at: https://www.mdpi.com/article/10.3390/biology15171556/s1, Table S1: Proximate composition of ML (% dry matter). Table S2: Formulation and compositions of experimental diets (%); Table S3: The complete calculation formula.; Table S4: Primer sequences for real-time PCR; Table S5: Effects of ML on the growth performance and morphometric parameters in *Acipenser schrenckii*.

## Abbreviations

The following abbreviations are used in this manuscript:

ML	Mulberry leaves
T-AOC	Total antioxidative capability
SOD	Superoxide dismutase
MDA	Malondialdehyde
C3	Complement 3
C4	Complement 4
IgM	Immunoglobulin M
DAO	Diamine oxidase
Vh	Villus height
Cd	Crypt depth
*tlr2-2*	Toll-like receptor 2
*tlr7*	Toll-like receptor 7
*myd88*	Myeloid differentiation primary response gene 88
*map3k14*	Mitogen-activated protein kinase kinase kinase 14
*nfκb1*	Nuclear factor kappa-B subunit 1
*cxcl13*	C-X-C motif chemokine ligand 13
*cxcl8*	C-X-C motif chemokine ligand 8
*keap1*	Kelch-like ECH-associated protein 1
*nrf2*	Nuclear Factor Erythroid 2-Related Factor 2
*mafk*	MAF bZIP Transcription Factor K
*cat*	Catalase
*sod1*	Cu,Zn-Superoxide Dismutase
*tnfα*	Tumor Necrosis Factor α
*il1β*	Interleukin 1β
*tgfβ*	Transforming Growth Factor β1
*il10*	Interleukin 10
