# Supplementary material for: Graded Mulberry Leaf Supplementation Shapes Gut Microbiota, Reprograms Intestinal Metabolism, and Maintains Intestinal Chemical–Immune Barrier Homeostasis in Amur Sturgeon: A Multi-Omics Study
Source: Biology (Basel). 2026 Sep 5;15(17):1556. doi: 10.3390/biology15171556 (PMC13564912; doi:10.3390/biology15171556)
Supplement: Supplementary file 1 [file biology-15-01556-s001.zip › biology-4531509-supplementary.pdf]

Supplementary Files

Table S1. Proximate composition of ML (% dry matter).

Ingredient	Dry matter	Crude fat	Crude protein	Crude ash	Crude fiber
ML	99.93	4.46	17.01	12.30	9.44

Table S2. Formulation and compositions of experimental diets (%).

Ingredients	CK	ML2	ML4	ML6
Fish meal[1]	55.00	54.45	53.35	52.25
ML	0.00	2.00	4.00	6.00
Wheat middling	18.00	15.67	10.35	5.00
Wheat gluten meal	19.00	19.00	22.00	25.00
Fish oil[1]	3.50	3.50	4.00	4.30
Soybean oil[1]	3.00	3.00	3.00	3.00
L-Lysine[2]	0.05	0.50	1.00	1.50
Yeast extract	1.00	1.00	1.00	1.00
DL-Met[2]	0.00	0.20	0.40	0.80
$Ca(H_2PO_4)_2$	0.05	0.08	0.10	0.15
Thr[2]	0.00	0.20	0.40	0.60
Mineral premix[3]	0.20	0.20	0.20	0.20
Vitamin premix[4]	0.20	0.20	0.20	0.20
Total	100	100	100	100
Analyzed nutrient composition (%, in dry matter basis)				
Dry matter	95.3	94.9	95.2	96.3
Crude ash	11.7	11.8	12.7	12.8
Crude protein	46.5	46.4	46.4	46.2
Crude lipid	11.4	11.2	11.6	11.9
Gross energy (MJ/kg)	19.7	19.5	19.4	20.0

[1] Fish meal was supplied by Pesquera Centinela Co., Lima, Peru. Fish oil and Soybean oil were

supplied by Han Ye Biotechnology Co.,Ltd. Shangdong, China.

[2] Referencing the ideal AA model of white sturgeon, calculated the supplementation level of CAA.

[3] Mineral premixes (mg/kg diet): $CuSO_4·5H_2O$ 10; $FeSO_4·H_2O$ 300; $ZnSO_4·H_2O$ 200; $MnSO_4·H_2O$ 80; KI (1%) 1.5; Na_2SeO_3 (10% Se) 10; $CoCl_2·6H_2O$ (10% Co) 20; Zeolite 378.5.

[4] Vitamin premix (mg/kg diet): Vitamin A 20; Vitamin B_1 12; Vitamin B_2 10; Vitamin B_6 15; Vitamin B_{12} (1%) 8; Niacinamide 100; Ascorbyl calcium phosphate (35%) 1000; Calcium pantothenate 40; Biotin (2%) 2; Folic acid 10; Vitamin E (50%) 400; Vitamin $_{K3}$ 20; Vitamin D_3 10; Inositol 200; Choline chloride (50%) 4000; Corn protein powder 150.

Table S3. The complete calculation formula.

Items	Calculation formula
WGR(%)	100×(Wt -Wo) /Wo
SGR(%/day)	100×[(ln(Wt)-ln(Wo)] /days of feed test
FCR	feed intake/(Wt -Wo + dead experimental fish weight)
FR(%)	100×total dry matter intake of feed/[(Wt+Wo)/2]/days of feed test
SR (%)	100×terminal quantity/original quantity
CF (g/cm^3)	100×(body weight) /[(body length)^3]
HSI (%)	100×(hepatic weight) / (body weight)
VSI (%)	100×(viscus weight) / (body weight)

Wt is the terminal body weight, Wo is the original body weight, the same below.

Table S4. Primer sequences for real-time PCR.

Gene	Sequence 5′-3′	Target size (bp)	Tm (°C)
βactin	F: CGGTTTCGCTGGAGATGATG	135	60

	R: TCAGTGAGCAGGACGGGGTG		
tlr2-2	F: GAACTTGTCAGCTGGTTGGT<br>R: AGAGCTGCCCATGACCTAAAG	94	58-59
tlr7	F: GCTGCAATTGCTCACACACT<br>R: GAGAGTTTTGGTGCAGTTGG	93	57-58
myd88	F: CTCTGACCTGCAAGGGCTTAT<br>R: ATCCCTGTGGCAGTAGCAGATG	202	59-60
map3k14	F: ACTCAGACACACATGGCACC<br>R: TCATGTGAAGCAGTGTGCAG	143	58-59
nfκb1	F: CAGGGGGAGAGGAGATGTAT<br>R: AGAGTGGGCCATTCTCATCT	92	57-58
cxcl13	F: GAGATGATTCCCAGAGGAGC<br>R: GGATCCATTTTGCTTCAGGG	99	57
cxcl8	F: CATCCCCAGAGGATCACACT<br>R: CCAGGGTGCACGAAGATTCA	90	58-59
tnfα	F: AAAATGTCCAGCCATCAAAGCA<br>R: GTGAGGAATGATCCCGTTGGT	132	60
il1β	F: TGGTGGAGAAGAGAACGCAAGATG<br>R: GCGTCACTGCCTGTCTCATGG	114	60
tgfβ	F: TGCTCCTGTGCTCGGCTCTC<br>R: CAGCTCCATGTCCAGAGTCTTGC	80	60
il10	F: GCAAAGCTGAGGGAGCTGAG<br>R: CCCAGATTGACAGCTGGAGG	194	60
keap1	F: CCAGACCGGATAACGAGCTGAATC<br>R: GCCTGGCTTGTGAGACTCTGTATC	101	60
nrf2	F: TGGAAGTGTCCAGCCTGAGT<br>R: GTCGGCAGTCCCTTCTTTGG	138	60
mafk	F: AAATCAGCCGACCACAAGAC<br>R: ACTGGATGGACTGGCAAGAG	161	58
cat	F: TGGACACCGACACATGAATGGC<br>R: GGCGATGGCGTTGTACAGGTC	190	60
sod1	F: ACTCCTGGAGAGCATGGCTT<br>R: GGGTTGAAGTGAGGACCAGC	83	60

Tm: melting temperature; F: forward primer; R: reverse primer.

Table S5. Effects of ML on the growth performance and morphometric parameters in *Acipenser schrenckii*.

Items	Groups				SEM	*P*-value		
	CK	ML2	ML4	ML6		ANOVA	Linear	Quadratic
**Growth performance**								
FBW(g)	421.05	427.05	407.35	406.75	6.32	0.1034		
SR	95	95	95	95	1.44	0.4262		
WGR(%)	112.48	113.99	105.51	104.74	2.80	0.0812		
SGR(%/d)	1.08	1.09	1.03	1.02	0.02	0.0781		
FCR	1.26^{ab}	1.21^{b}	1.33^{a}	1.33^{a}	0.03	0.0212	0.0304	0.4554
FR(%)	1.28	1.28	1.30	1.32	0.01	0.0868		
**Morphometric parameters**								
CF(g/cm^3)	0.76	0.75	0.74	0.75	0.02	0.2442		
HSI(%)	3.77^{a}	3.39^{ab}	3.44^{ab}	2.92^{b}	0.16	0.0245	0.0019	0.6714
VSI(%)	10.22	10.23	10.2	9.82	0.38	0.8727		

FBW = final body weight; SR = survival rate; WGR= weight gain rate; SGR = specific growth rate; FCR = feed conversion rate; FR = feeding rate; CF = condition factor; HSI = hepatosomatic index; VSI = viscerosomatic index.

$^{a-b}$ Within a row, means without a common superscript letter differ at $P < 0.05$, $n = 4$.
